# Site-Selective d^10^/d^0^ Substitution in an *S* = ^1^/_2_ Spin Ladder Ba_2_CuTe_1–*x*_W_*x*_O_6_ (0 ≤ *x* ≤ 0.3)

**DOI:** 10.1021/acs.inorgchem.1c03655

**Published:** 2022-02-21

**Authors:** Charlotte Pughe, Otto H. J. Mustonen, Alexandra S. Gibbs, Martin Etter, Cheng Liu, Siân E. Dutton, Aidan Friskney, Neil C. Hyatt, Gavin B. G. Stenning, Heather M. Mutch, Fiona C. Coomer, Edmund J. Cussen

**Affiliations:** † Department of Material Science and Engineering, University of Sheffield, Sheffield S1 3JD, United Kingdom; ‡ School of Chemistry, University of Birmingham, Edgbaston, Birmingham B15 2TT, United Kingdom; § School of Chemistry, University of St Andrews, North Haugh, St Andrews KY16 9ST, United Kingdom; ∥ ISIS Pulsed Neutron and Muon Source, STFC Rutherford Appleton Laboratory, Didcot OX11 0QX, United Kingdom; ⊥ Max Planck Institute for Solid State Research, Heisenbergstrasse 1, 70569 Stuttgart, Germany; # Deutsches Elektronen-Synchrotron (DESY), 22607 Hamburg, Germany; @ Cavendish Laboratory, University of Cambridge, J. J. Thomson Avenue, Cambridge CB3 0HE, United Kingdom; ∇ Johnson Matthey Battery Materials, Reading RG4 9NH, United Kingdom

## Abstract

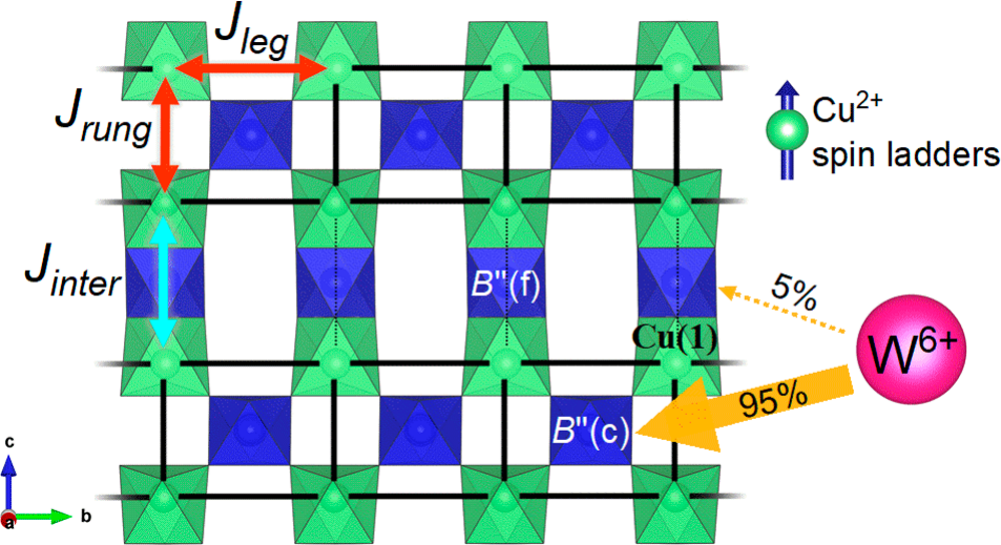

Isovalent nonmagnetic d^10^ and d^0^ B″ cations have proven to be a powerful tool for tuning the magnetic interactions between magnetic B′ cations in A_2_B′B″O_6_ double perovskites. Tuning is facilitated by the changes in orbital hybridization that favor different superexchange pathways. This can produce alternative magnetic structures when B″ is d^10^ or d^0^. Furthermore, the competition generated by introducing mixtures of d^10^ and d^0^ cations can drive the material into the realms of exotic quantum magnetism. Here, Te^6+^ d^10^ was substituted by W^6+^ d^0^ in the hexagonal perovskite Ba_2_CuTeO_6_, which possesses a spin ladder geometry of Cu^2+^ cations, creating a Ba_2_CuTe_1–*x*_W_*x*_O_6_ solid solution (*x* = 0–0.3). We find W^6+^ is almost exclusively substituted for Te^6+^ on the corner-sharing site within the spin ladder, in preference to the face-sharing site between ladders. The site-selective doping directly tunes the intraladder, *J*_rung_ and *J*_leg_, interactions. Modeling the magnetic susceptibility data shows the d^0^ orbitals modify the relative intraladder interaction strength (*J*_rung_/*J*_leg_) so the system changes from a spin ladder to isolated spin chains as W^6+^ increases. This further demonstrates the utility of d^10^ and d^0^ dopants as a tool for tuning magnetic interactions in a wide range of perovskites and perovskite-derived structures.

## Abstract 2

Substitution of d^10^ or d^0^ cations modifies the magnetic interactions in perovskites due to their differing contributions to superexchange. Here, we demonstrate the d^10^/d^0^ effect can be site-selectively applied to the corner-sharing site in a hexagonal perovskite, Ba_2_CuTe_1−*x*_W_*x*_O_6_. This directly tunes the intraladder interactions in the Cu^2+^ spin ladder and modifies the magnetic behavior. This further demonstrates the utility of the d^10^/d^0^ effect for modifying magnetic interactions in perovskites.

## 1 Introduction

Chemical doping is widely used to tune, control, and influence the properties of materials. The periodic table offers a plethora of dopants from which to choose on the basis of differences in charge and ionic radii. By careful selection, it is possible to desirably modify the structural, electronic, and magnetic properties and, in some cases, to generate behaviors entirely different from those of the parent compound. The classic example is Sr^2+^ doping of the antiferromagnetic layered perovskite-type La_2_CuO_4_ that leads to high *T*_C_ superconductivity in La_2–*x*_Sr_*x*_CuO_4_ (*x* = 0.06–0.25).^1−3^ This discovery has fascinated scientists for decades and led to a cascade of studies investigating low-dimensional copper systems.

**Figure 1:**
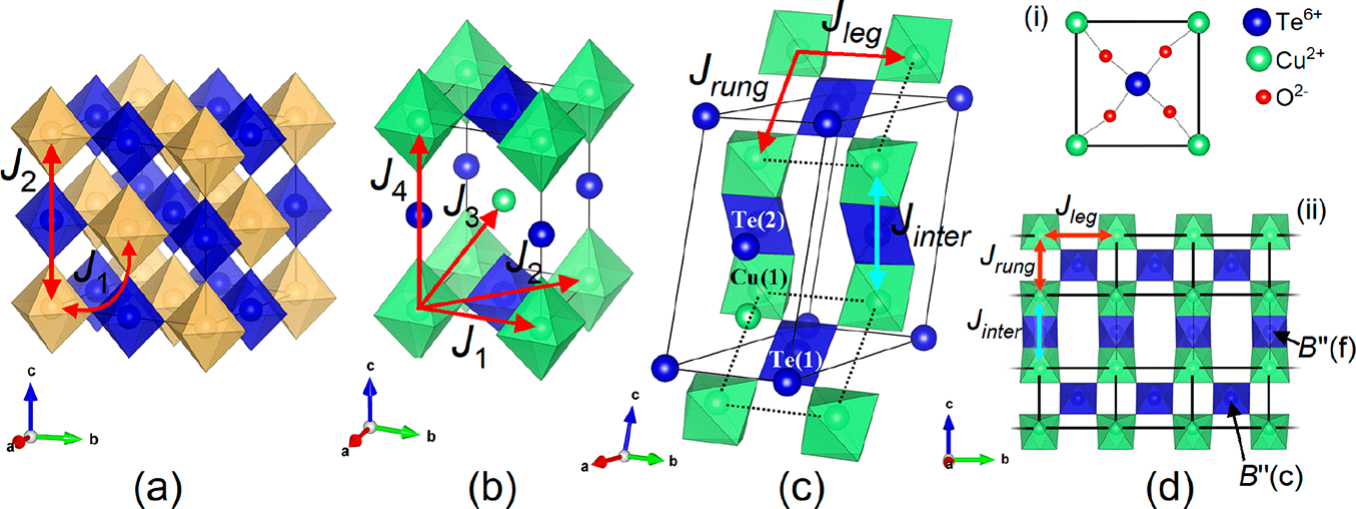
Magnetic interactions in B″ = W^6+^ (d^0^) and/or Te^6+^ (d^10^) perovskite structures. (a) Simple fcc Heisenberg *J*_1_ and *J*_2_ interactions in the cubic double perovskites Ba_2_Mn(Te/W)O_6_. (b) Heisenberg square lattice interactions in the Sr_2_Cu(Te/W)O_6_, Ba_2_CuWO_6_, and Ba_2_CuTeO_6_ high-pressure tetragonal perovskites. (c) Spin ladder interactions in the hexagonal perovskite Ba_2_CuTeO_6_. (d) (i) Structural motif present in the Sr_2_Cu(Te/W)O_6_ and Ba_2_CuTeO_6_ structures. The structural motif consists of four corner Cu^2+^ cations interacting via Cu–O–B″–O–Cu superexchange. (d) (ii) Illustration of the Cu^2+^ spin ladders running along the *b*-axis within the Ba_2_CuTeO_6_ structure when viewed along the *a–b* plane. The intraladder (*J*_leg_ and *J*_rung_) and interladder (*J*_inter_) interactions are shown by the red and blue arrows, respectively. The corner-sharing B″(c) and face-sharing B″(f) Te^6+^ sites in hexagonal Ba_2_CuTeO_6_ are indicated by the black arrows.

Dopants have such dramatic effects because they intrinsically modify the interactions within the parent material. In magnetic oxides, these interactions are typically superexchange interactions mediated by oxygen anions. These interactions are generally well understood when the magnetic cations are connected by a single oxygen anion.^4^ However, the situation is more complicated when the magnetic cations are farther away and the interactions occur by extended superexchange. Recently, a new method for directly tuning these extended superexchange interactions has been developed.^5,6^ This method is based on doping diamagnetic d^10^ and d^0^ cations into extended superexchange pathways that link magnetic cations. This d^10^/d^0^ effect can be used in A_2_B′B″O_6_ double perovskites, where B′ is a magnetic cation and B″ is a diamagnetic d^10^ or d^0^ cation.^7,8^ The double perovskite structure consists of corner-sharing B′O_6_ and B″O_6_ octahedra alternating in a rock salt-type order (Figure 1a).^9^ The superexchange between the magnetic B′ cations is extended via orbital overlap with the linking B″ cations and O 2p orbitals (i.e., B′–O–B″–O–B′).

We have recently shown that diamagnetic d^10^ and d^0^ cations on the linking B″ site have a significant effect on the magnetic interactions and ground states in double perovskites.^7,10^ We investigated this d^10^/d^0^ effect in the cubic double perovskites Ba_2_MnTeO_6_ and Ba_2_MnWO_6_, in which the magnetic Mn^2+^ cations are linked by either 4d^10^ Te^6+^ or 5d^0^ W^6+^ B″ cations. In these isostructural materials, Mn^2+^*S* = ^5^/_2_ magnetism is described using a simple face-centered cubic (fcc) Heisenberg model consisting of a 90° [nearest neighbor (NN), *J*_1_] and 180° [next-nearest neighbor (NNN), *J*_2_] Mn–O–(Te/W)–O–Mn interaction (Figure 1a). Neutron scattering experiments demonstrated the dominant interaction strongly depends on the nonmagnetic B″ cation, with a stronger *J*_1_ when B″ = Te^6+^ (4d^10^) and a stronger *J*_2_ when B″ = W^6+^ (5d^0^). The contrasting *J*_1_ and *J*_2_ interactions produce entirely different magnetic structures for Ba_2_MnTeO_6_ (type I AFM) and Ba_2_MnWO_6_ (type II AFM).

The d^10^/d^0^ effect is caused by differences in orbital hybridization in the B′–O–B″–O–B′ superexchange pathways. When B″ = Te^6+^, there is no d-orbital contribution to superexchange as the 4d^10^ orbitals lie far below the Fermi level.^11^ Therefore, the majority of superexchange occurs via a NN B′–O–O–B′ interaction.^12,13^ Conversely, when B″ = W^6+^, the 5d^0^ orbitals strongly hybridize with O 2p allowing W^6+^ to directly contribute to extended superexchange via NNN B′–O–W^6+^–O–B′.^14^ This effect limits the NNN *J*_2_ exchange in Te^6+^ compounds, as this superexchange pathway requires a d-orbital contribution from the B″ cation. We also highlight the fact that the d^10^/d^0^ effect extends beyond simple cubic structures to a large range of 3d transition metal B′ *=* Co,^15,16^ Ni,^17−19^ and Cu^11,14,20,21^ double perovskites, all of which follow the same principle based on the nonmagnetic B″ site: d^0^ with strong *J*_2_ (type II) or d^10^ with strong *J*_1_ (type I/Néel order).

The most striking examples of the d^10^/d^0^ effect in 3d double perovskites are the Cu^2+^*S* = ^1^/_2_ compounds Sr_2_CuTeO_6_ and Sr_2_CuWO_6_ and their solid solution Sr_2_CuTe_1–*x*_W_*x*_O_6_, where the d^10^/d^0^ doping stabilizes a novel quantum disordered ground state. Here, the combination of the Cu^2+^ Jahn–Teller (J–T) effect and orbital ordering produces a square lattice Heisenberg antiferromagnet, with highly two-dimensional magnetism.^13,21,22^ The tetragonal unit cell has square lattice *a–b* planes of Cu^2+^ cations in which superexchange is described using in-plane *J*_1_ (NN) and *J*_2_ (NNN) interactions, but with additional weak interplane interactions (*J*_3_ and *J*_4_) along *c* (Figure 1b).^11,21^ Following the principles of the d^10^/d^0^ effect, Sr_2_CuTeO_6_ is Néel ordered, while a strong *J*_2_ leads to columnar ordering for Sr_2_CuWO_6_.^13,14,20,23−25^ Using the d^10^/d^0^ effect by making a Sr_2_CuTe_1–*x*_W_*x*_O_6_ solid solution allows for the direct tuning of magnetic interactions on the square lattice between the strong *J*_1_ (*x* = 0) and strong *J*_2_ (*x* = 1) limits.^8,26^ The d^10^/d^0^ substitution results in the strong suppression of magnetic order as a quantum disordered ground state is observed for a wide composition range of *x* = 0.05–0.6.^8,12,26−29^ The 50:50 mixture Sr_2_CuTe_0.5_W_0.5_O_6_ closely resembles a quantum spin liquid, an exotic magnetic state in which the moments remain dynamic at 0 K and have been highly sought since they were first proposed in the 1970s.^30−33^

The question of whether d^10^/d^0^ doping can be used to tune magnetic interactions and induce exotic magnetic states in other magnetic lattices than the square lattice remains, and whether this can be extended from perovskites to perovskite-derived structures. Depending on the choice of A and B′/B″ cations, B′–O–B″–O–B′ linkers form between corner-sharing or/and face-sharing octahedra, generating the classic double perovskite structure in the purely corner-sharing case, while the introduction of face sharing creates the hexagonal perovskite structure.^34−36^ The observation of tunable magnetic interactions in structures with different octahedral connectivity would suggest d^10^/d^0^ substitutions can be employed in a range of materials to access novel quantum states, many of which are hard to realize experimentally.^37^

Ba_2_CuTeO_6_ is an excellent system for testing this due to its hexagonal perovskite structure that results in a spin ladder magnetic geometry.^38−41^ Within the spin ladder, Cu^2+^ cations are linked via three key Cu–O–Te–O–Cu exchange interactions illustrated in Figure 1c. These are the intraladder *J*_leg_ and *J*_rung_ interactions via the corner-sharing Te(1)O_6_ units and the interladder interaction via the face-sharing Te(2)O_6_ units within the Cu–Te(2)–Cu trimers.^42^ The intraladder interactions are antiferromagnetic and equally strong with *J*_rung_/*J*_leg_ ∼ 1, while the interladder interaction is weaker.^38,39^ In principle, W^6+^ could be doped onto either of the Te^6+^ B″ sites. This offers the possibility of tuning the *J*_leg_ and *J*_rung_ interactions independently of the *J*_inter_, forming a more complex phase space than cubic perovskites. For clarity, the two B″ sites are henceforth labeled B″(c) and B″(f), where c and f denote corner and face sharing, respectively. The B″(c) and B″(f) sites are indicated in Figure 1d(ii), which shows the Cu^2+^ spin ladders running along the *b*-axis of the Ba_2_CuTeO_6_ structure. The intraladder interactions in Ba_2_CuTeO_6_ are quite similar to the Cu–O–Te–O–Cu interactions within the square lattice of Sr_2_CuTeO_6_. Both structures share the structural motif shown in Figure 1d(i) involving four corner Cu^2+^ cations interacting via Cu–O–B″–O–Cu superexchange. In addition, the significant *J*_inter_ leads to the formation of a Néel ordered ground state for Ba_2_CuTeO_6_, the same type of ordering observed for Sr_2_CuTeO_6_.^39^ Hence, in a manner analogous to that of Sr_2_CuTe_1–*x*_W_*x*_O_6_, one might expect similar strong suppression of magnetic order upon site-specific doping of B″ d^0^ cations onto the intraladder B″(c) sites in Ba_2_CuTe_1–*x*_W_*x*_O_6_.^26,28,29^

To answer these questions, we prepared the Ba_2_CuTe_1–*x*_W_*x*_O_6_ solid solution (0 ≤ *x* ≤ 0.3). Using a combination of crystallographic and spectroscopic techniques, we show that W^6+^ can be site-selectively doped onto the corner-sharing B″(c) site in Ba_2_CuTe_1–*x*_W_*x*_O_6_. This site selectivity allows for the direct tuning of intraladder interactions, which show a strong decrease in *J*_rung_/*J*_leg_ with an increase in *x*. Our work demonstrates the d^10^/d^0^ effect can be extended to perovskite-derived structures such as hexagonal perovskites.

## 2 Experimental Section

### 2.1 Synthesis

Conventional solid-state chemistry techniques were used to synthesize polycrystalline samples of Ba_2_CuTe_1–*x*_W_*x*_O_6_. The *x* = 0, 0.05, 0.1, 0.2, and 0.3 compositions were prepared by thoroughly mixing stochiometric quantities of high-purity BaCO_3_ (99.997%), CuO (99.9995%), TeO_2_ (99.995%), and WO_3_ (99.998%) (all purchased from Alfa Aesar) in an agate mortar. The reactant mixtures were pelletized and calcined at 900 °C in air, before being fired at 1000–1100 °C for 24 h periods with intermittent grinding. The phase purity was monitored using X-ray diffraction (Rigaku Miniflex, Cu Kα). A total of 72–120 h was required to achieve phase purity in all compositions, with the heating time increasing as the W content increased. The synthesis was stopped once single-phase samples were obtained. The sample color changed from yellow to a darker yellow-brown across the solution from *x* = 0 to *x* = 0.3, which may be indicative of a gradual modification of the band gap as the W^6+^ content increased.

### 2.2 Magnetization and Heat Capacity Measurements

Magnetic characterization was performed using a Quantum Design MPMS3 magnetometer (Magnetic Property Measurement System). Approximately 100 mg of powder was sealed in a gelatin capsule, which was then secured in a polymer straw sample holder. Zero-field-cooled (ZFC) and field-cooled (FC) curves were measured between 2 and 300 K in dc superconducting quantum interference device mode using an external field of 0.1 T. ac measurements were taken between 2 and 100 K using a dc field of 25 Oe and an ac field of 5 Oe using ac frequencies between 10 and 467 Hz. Heat capacity measurements were performed using a Quantum Design Physical Property Measurement System instrument. The samples were mixed with silver (99.999%) in a 1:1 gravimetric ratio to enhance the low-temperature thermal conductivity. The Ba_2_CuTe_1–*x*_W_*x*_O_6_:Ag powder was pressed into a pellet. The pellet was broken, and ∼10 mg shards were selected; the heat capacity was measured between 2 and 120 K using the thermal relaxation method. The silver contribution was removed on the basis of a measurement of pure silver powder.

### 2.3 Neutron Powder Diffraction

The nuclear structure of *x* = 0.05, 0.1, and 0.3 compounds was investigated using the High Resolution Powder Diffractometer (HRPD) at the ISIS Neutron and Muon Source. Approximately 8 g of each sample powder was loaded into an Al-alloy slab-can and sealed using vanadium windows. The exposed surfaces of the slab-can were shielded using highly absorbing Gd and Cd foils so that only the vanadium windows of the can were exposed to the neutron flux. After the slab-can had been aligned perpendicular to the neutron beam, time-of-flight neutron powder diffraction patterns were recorded between 2 and 300 K using a cryostat to cool the sample. The data can be found online.^43^ The data were corrected for sample absorption, and Rietveld refinements were performed using GSAS-2.^44,45^ VESTA was used to visualize the crystal structures.^46^

### 2.4 Synchrotron X-ray Diffraction

The *x* = 0.1, 0.2, and 0.3 samples were loaded into 0.6 mm diameter glass capillaries and measured at room temperature on the P02.1 beamline at the PETRA III X-ray radiation source (DESY) using a wavelength λ of 0.20742 Å. The capillary was located 1169.45 mm from the PerkinElmer XRD1621 two-dimensional (2D) detector and spun during the measurement. The 2D data were processed using DAWN Science. The one-dimensional data obtained were refined using GSAS-2. The X-ray and neutron data (as well as the bulk magnetization data) were collected using samples from the same batch.

### 2.5 Extended X-ray Absorption Fine Structure (EXAFS)

EXAFS measurements were performed on the Beamline for Materials Measurement (6-BM) at National Synchrotron Light Source II (NSLS-II). Room-temperature X-ray absorption spectra (XAS) of the *x* = 0.3 compound were recorded in transmission mode near the W L_3_ edge, using a finely ground specimen dispersed in polyethylene glycol to achieve a thickness of one absorption length. Incident and transmitted beam intensities were measured using ionization chambers, filled with mixtures of He and N_2_, operated in a stable region of their *I*/*V* curve. A tungsten foil was used as an internal energy calibration where the first inflection point in the measured W L_3_ edge was defined to be *E*_0_ = 10206.8 eV. Data reduction and analysis were performed using Athena, Artemis, and Hephaestus.^47^ The EXAFS data were then analyzed using ATOMS and FEFF in the Artemis package with the monoclinic structural model.

## 3 Results

### 3.1 Crystal Structure

Initial structural characterization was achieved using laboratory X-ray diffraction. The laboratory X-ray diffraction (XRD) patterns confirm phase purity from *x* = 0 to *x* = 0.3. Attempts were made to synthesize richer W^6+^ samples, but beyond *x* = 0.3, significant W^6+^ impurities formed. These impurities were not diminished on further heating, showing the *x* = 0.3 composition lies close to the solubility limit for our synthesis procedure. Rietveld refinement showed all of the Ba_2_CuTe_1–*x*_W_*x*_O_6_ structures adopt the same *C*2/*m* symmetry as the Ba_2_CuTeO_6_ parent structure. The unit cell volume decreases linearly with *x* (see the inset of Figure S1); this showing Vegard’s law behavior indicates successful doping of W^6+^ into the Ba_2_CuTeO_6_ structure. Synchrotron X-ray diffraction, EXAFS, and neutron diffraction studies provided further insight into the structural effects of doping across the solution.

#### 3.1.1 Synchrotron X-ray Diffraction

**Figure 2:**
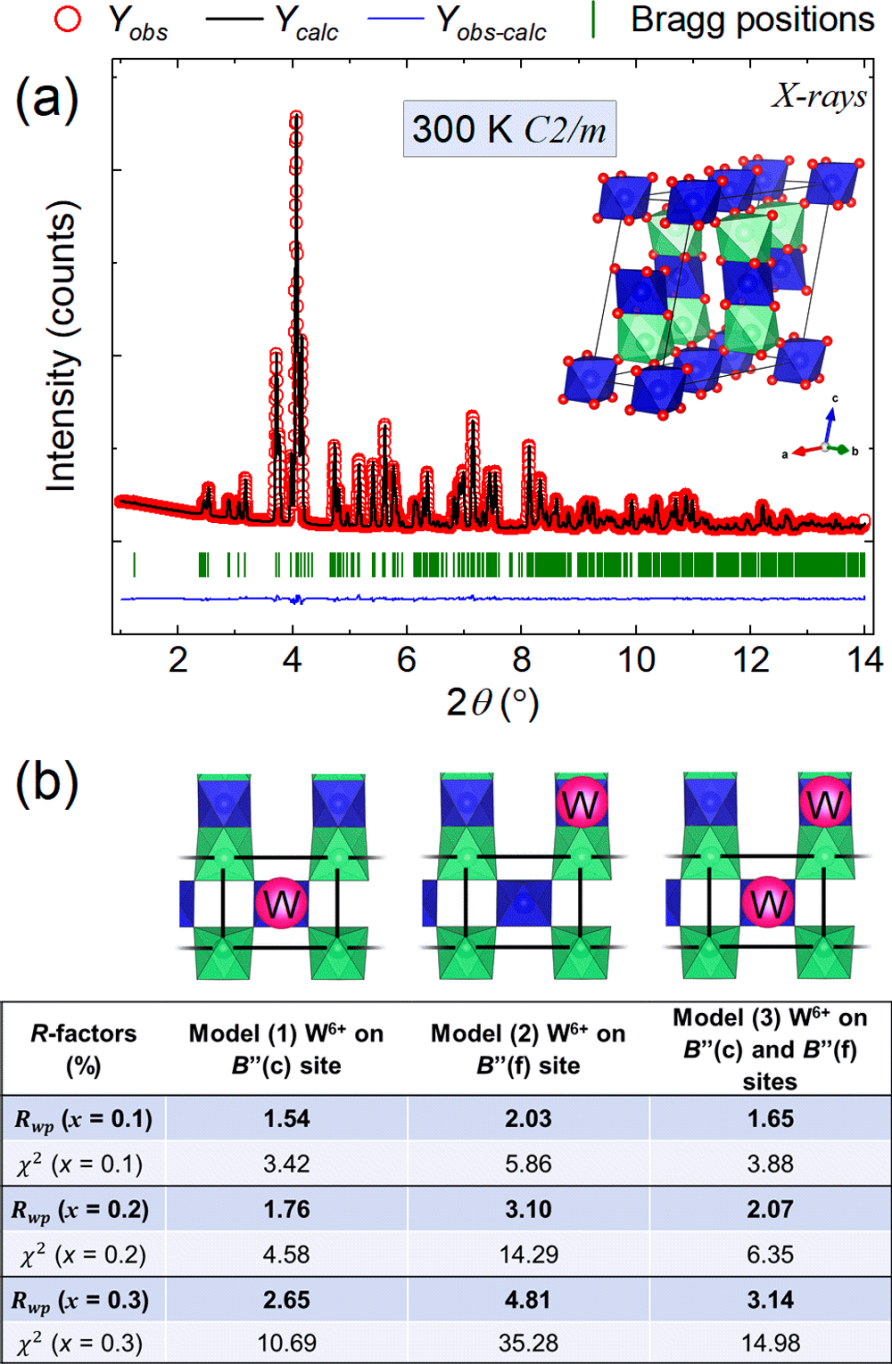
(a) Synchrotron X-ray diffraction pattern of Ba_2_CuTe_0.8_W_0.2_O_6_ at room temperature collected using a wavelength λ of 0.20742 Å. (b) *R*-Factors obtained from Rietveld refinement using the three different W^6+^ site occupancy models for Ba_2_CuTe_1–*x*_W_*x*_O_6_. The crystal structures directly above the *R*-factors for each model depict the placement of W^6+^ (pink) on either the corner-sharing B″(c) site, the face-sharing B″(f) site, or both the B″(c) and B″(f) sites (50:50) in the *x* = 0.1, 0.2, and 0.3 compositions. The Te^6+^ cations are colored blue, and the Cu^2+^ cations are colored green in the spin ladder.

Figure 2a shows an illustrative synchrotron X-ray diffraction pattern collected for the *x* = 0.2 sample. Data collected at 300 K for *x* = 0.1, 0.2, and 0.3 compounds were all used to test three possible site occupancy models. These models are (1) W^6+^ exclusively on the B″(c) site, (2) W^6+^ exclusively on the B″(f) site, and (3) W^6+^ occupying both B″(c) and B″(f) sites. An equal distribution (50:50) was initially assumed in model 3. The results in panels a and b of Figure 2 show model 1 reproduces the observed diffraction profile uniquely well. Figure 2b shows *R*_wp_ is consistently lower when W^6+^ exclusively occupies the B″(c) site in *x* = 0.1, 0.2, and 0.3 compounds. This suggests a strong preference for corner-sharing, which was further evaluated by allowing the site occupancies to be refined, within the constraints of sample stoichiometry. This identified a small amount of W^6+^ on the B″(f) site in each sample, with the site occupancy increasing linearly with *x* to a maximum value of ∼3% as shown in Table 1. In each refinement, 5% of the W^6+^ present in the sample is found on the face-sharing octahedral site. Comparing the *R*_wp_ and χ^2^ values in Table 1 to those in Figure 2b shows minor occupation of the B″(f) site by W^6+^ leads to a slight but not negligible improvement in the fit compared to model 1. W^6+^ occupancy of the B″(f) site was confirmed by refinements using the initial site occupancies from model 1 and model 2 as starting values. Both refinements converged to the same results in Table 1. Given the energetics for ion migration diminishes on cooling from room temperature, the site preference undoubtedly extends to the low-temperature structures.

**Table 1:** Refined B″(c) and B″(f) Site Fractions Determined Using the *x* = 0.1, 0.2, and 0.3 Synchrotron X-ray Diffraction Data^a^

	B″(c)		B″(f)				
	Te(1)	W(1)	Te(2)	W(2)	percentage of total W^6+^ on the B″(f) site	*R*_wp_ (%)	χ^2^
*x* = 0.1	0.809(1)	0.191(1)	0.991(1)	0.009(1)	4.7(2)	1.54	3.39
*x* = 0.2	0.618(1)	0.382(1)	0.982(1)	0.018(1)	4.7(2)	1.75	4.54
*x* = 0.3	0.430(1)	0.570(1)	0.970(1)	0.030(1)	5.3(2)	2.60	10.50^b^

a The W(1) and W(2) site fractions were used to calculate the percentage of the total amount of W^6+^ on the B″(f) site in each composition. Also shown are the *R*_wp_ and χ^2^ values for the Rietveld fits.

b The larger χ^2^ for the *x* = 0.3 composition reflects a longer counting time compared to those of the *x* = 0.1 and 0.2 samples.

#### 3.1.2 Extended X-ray Absorption Fine Structure (EXAFS)

**Figure 3:**
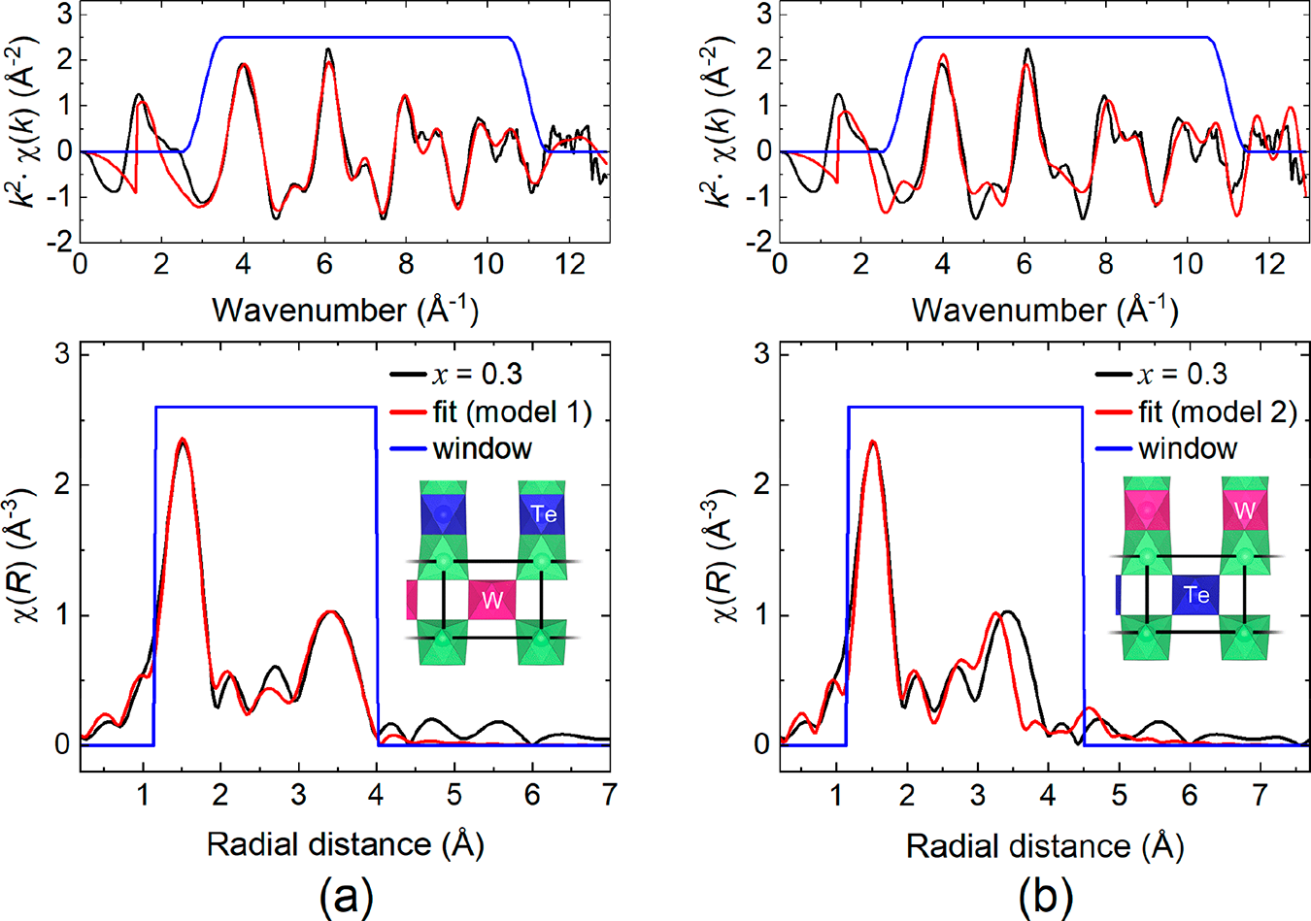
(a) *k*^2^χ(*k*) and χ(*R*) W L_3_ EXAFS data of Ba_2_CuTe_0.7_W_0.3_O_6_ with model 1, assuming W^6+^ doping on the B″(c) site (uncorrected for phase shift). (b) *k*^2^χ(*k*) and χ(*R*) W L_3_ EXAFS data of Ba_2_CuTe_0.7_W_0.3_O_6_ with model 2, assuming W^6+^ doping on the B″(f) site (uncorrected for phase shift). In panels a and b, the solid black lines represent the experimental data and the red lines represent the model fits. Fitting windows are indicated by solid blue lines. The crystal structures in the plots of χ(*R*) vs radial distance depict the models used in the fits. W^6+^ on the B″(c) site in model 1 and the B″(f) site in model 2 is colored pink, while the Te^6+^ cations are colored blue. The Cu^2+^ cations in the spin ladder are colored green.

Analysis of W L_3_ EXAFS data considered the following models, (1) full W^6+^ substitution on the B″(c) site and (2) full W^6+^ substitution on the B″(f) site, within the monoclinic structure. Model 1 afforded a plausible W^6+^ environment at the B″(c) site, with reasonable path lengths and positive Debye–Waller factors (Table S11). Figure 3a shows an excellent fit to the data, with an *R*-factor of 1.18%. In contrast, model 2 did not afford a plausible W^6+^ environment at the B″(f) site, with several paths having negative Debye–Waller factors (Table S12). The fit for model 2 in Figure 3b shows obvious regions of poor fit when compared to model 1 in Figure 3a and has a comparatively higher *R*-factor of 9.07%. This is because W^6+^ substitution at the B″(f) site does not provide adequate scattering paths to fit the significant second-shell contribution observed in the χ(*R*) transform of the EXAFS data in the *R* range of 3–4 Å (compare Figures S18 and S20). This supports the strong preference for W^6+^ doping at the B″(c) site, in agreement with the synchrotron X-ray data, but has the added advantage of providing an element-specific perspective. Attempts were made to fit the EXAFS data using contributions from both models 1 and 2, under a linear constraint, to assess the potential for disorder of a fraction of W^6+^ from the B″(c) to B″(f) site. However, it was not possible to adequately stabilize such a fit, because the number of variables approached the number of data points.

#### 3.1.3 Neutron Diffraction

**Figure 4:**
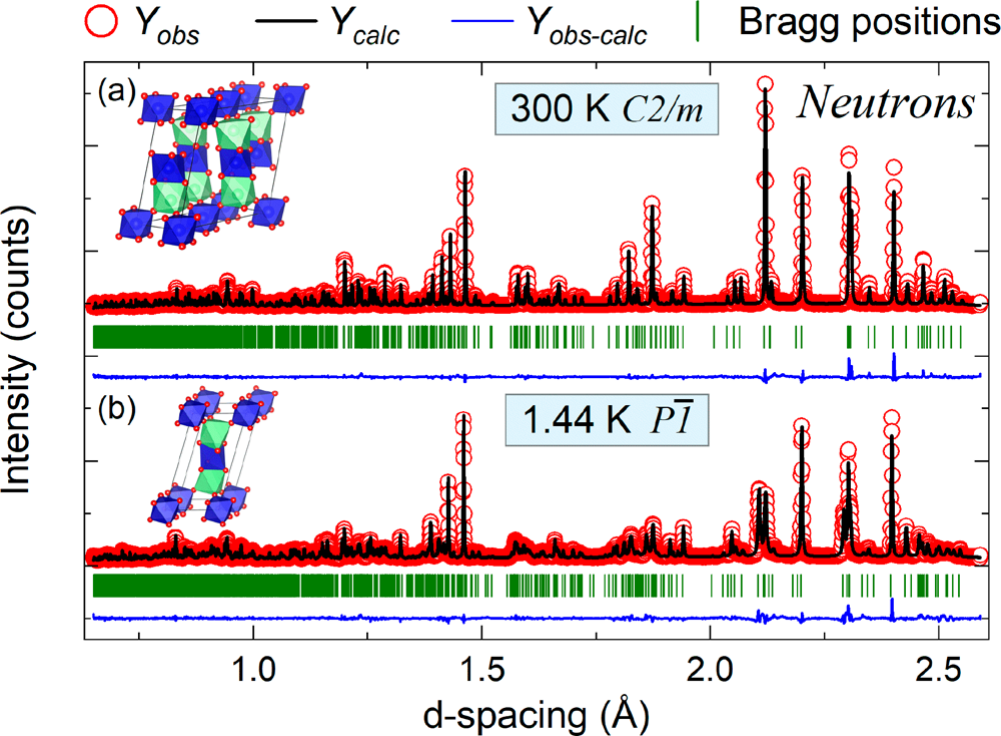
Neutron diffraction data showing the high-resolution powder diffraction (HRPD) patterns of Ba_2_CuTe_0.7_W_0.3_O_6_ at (a) 300 K and (b) 1.44 K.

In low-dimensional systems, the most striking quantum magnetic behavior may emerge at low temperatures.^48,49^ Consequently, variable-temperature neutron diffraction studies were performed on *x* = 0.1 and *x* = 0.3 compounds between 2 and 300 K to identify the low-temperature structure across the series. Both *x* = 0.1 and *x* = 0.3 compounds undergo the same *C*2/*m* to *P*1 transition as Ba_2_CuTeO_6_ on cooling.^42^ The transition *T*_trans_ was marked by peak splitting, as one can observe by comparing the neutron diffraction patterns of Ba_2_CuTe_0.7_W_0.3_O_6_ at (a) 300 K and (b) 1.44 K in Figure 4. As *x* in Ba_2_CuTe_1–*x*_W_*x*_O_6_ increased, peak splitting was suppressed to lower temperatures. Where *T*_trans_ is just below room temperature (287 K) for Ba_2_CuTeO_6_,^42^ comparing the *R*-factors from refinements using the *C*2/*m* and *P*1 models places *T*_trans_ between 235 and 240 K in the *x* = 0.1 compound and 100–120 K in the *x* = 0.3 compound. The decrease in *T*_trans_ is expected to follow across the series down to the minimum at *x* = 0.3 as the level of cation disorder increases with *x*. Importantly, it is clear the low-temperature structure at 2 K is triclinic.

The transition from *C*2/*m* to *P*1 is caused by weak symmetry breaking arising from further J–T distortion of the CuO_6_ octahedra on cooling. The structural integrity of the 12R hexagonal stacking sequence is retained upon the transition to lower symmetry. Like in the *C*2/*m* structure, extended Cu–O–B″–O–Cu superexchange occurs via the corner-sharing B″(c)O_6_ and face-sharing B″(f)O_6_ units forming the intraladder and interladder exchange interactions depicted in Figure 1c. While there are changes in the bond lengths and angles, the spin ladder structure can be regarded as almost the same in both the room-temperature monoclinic and the low-temperature triclinic structure. Given the close similarity, the *C*2/*m* structure has been previously used to model the low-temperature magnetic interactions as the higher symmetry simplifies the calculations.^39^

The dependence of temperature on the CuO_6_ octahedra was measured empirically using the J–T distortion parameter (*σ*_JT_) in eq 1.^50,51^
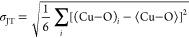
1where (Cu–O)_*i*_ represents the Cu–O neutron bond length on the *i*-Cu(1)O_6_ site and ⟨Cu–O⟩ is the mean bond length. *σ*_JT_ is plotted as a function of *T* for *x* = 0.1 and *x* = 0.3 compounds in Figure S15. As expected, *σ*_JT_ was found to be large and non-zero, reflecting uneven elongation of the axial Cu–O bonds to accommodate both corner and face sharing with the B″O_6_ octahedra. *σ*_JT_ gradually increased with a decrease in temperature for both samples, with the increasing distortion driving the transition to *P*1 symmetry. It appears the CuO_6_ octahedra in the *x* = 0.3 sample are slightly less distorted, possibly due to differences in covalency between W^6+^ and Te^6+^. However, compared to *x* = 0.1, the difference in *σ*_JT_ is minor and both samples plateau to the same distortion limit at 100 K.

The similarity in the neutron scattering lengths of Te and W, and the similarity of the preferred coordination site of these cations, meant it was not possible to determine the B″(c) versus B″(f) site distribution from the neutron diffraction data. Therefore, the B″(c) and B″(f) site occupancies determined from the synchrotron X-ray diffraction data were used in the crystal structural models.

### 3.2 Bulk Characterization

#### 3.2.1 Magnetic Susceptibility

**Figure 5:**
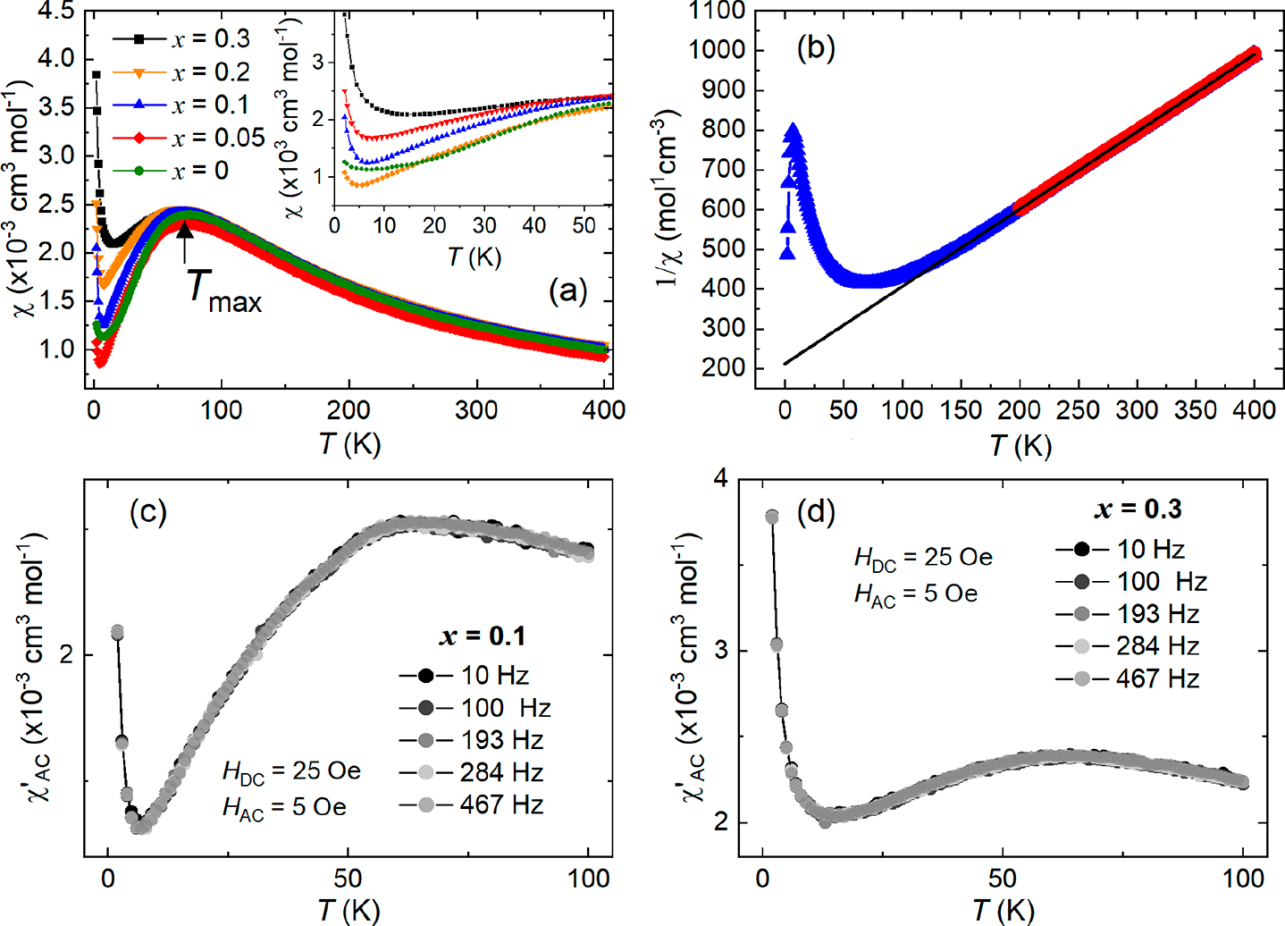
(a) dc magnetic susceptibility data for Ba_2_CuTe_1–*x*_W_*x*_O_6_ (*x* = 0–0.3) measured using an external field of 0.1 T. ZFC curves are shown as a function of temperature *T* between 2 and 400 K. The inset shows an expansion of the low-temperature χ vs *T* curve, where the Curie-tail-like features are observed for the W^6+^-doped samples. (b) Example Curie–Weiss fit of the 1/χ vs *T* data for Ba_2_CuTe_0.9_W_0.1_O_6_ between 200 and 400 K. (c and d) ac susceptibility curves for *x* = 0.1 and *x* = 0.3, respectively. The *χ′*_ac_ vs *T* data show no frequency dependence with ac frequency between 10 and 467 Hz.

dc magnetic susceptibility data collected for *x* = 0, 0.05, 0.1, 0.2, and 0.3 compounds using a field of 0.1 T are shown in Figure 5a. The curve of χ versus *T* of *x* = 0 is identical to previous reports.^38,40^ Upon cooling, there is a broad maximum at a *T*_max_ of ∼74 K, corresponding to short-range ladder interactions. Below *T*_max_, the susceptibility decreases before leading onto a small upturn beyond 14 K. The low-temperature behavior is believed to be indicative of the departure from ladder behavior and entry to the long-range ordered Néel state.^38^ However, the low-dimensional magnetic behavior means the system does not present a classical AFM ordering cusp, and the low-temperature upturn is not a general sign of magnetic order. Instead, magnetic order has been detected using muon and inelastic neutron scattering techniques placing the magnetic transition at a *T*_N_ of 14 K.^39,41^ It is likely to be coincidental that the upturn occurs close to the *T*_N_ of Ba_2_CuTeO_6_.

The W^6+^-doped samples are similar in that they all display the same broad *T*_max_ feature, but the position of *T*_max_ shifts to lower temperatures as *x* increases as shown in Table 2, suggesting weakening of the short-range ladder interactions. More dramatic differences are observed at low temperatures, where the upturn in the susceptibility data gradually becomes stronger with *x* creating a “Curie-tail”-like feature that is most pronounced for the *x* = 0.3 sample. These Curie-tail features show no field dependence upon measurement at higher external fields of 1 T.

**Table 2:** Results from Analysis of dc Magnetic Susceptibility Curves

	*x* = 0	*x* = 0.05	*x* = 0.1	*x* = 0.2	*x* = 0.3
*T*_max_ (K)	73.9	72.3	70.5	67.1	63.8
*C* (cm^3^ K mol^–1^)	0.5018(4)	0.4582(3)	0.5189(2)	0.5450(5)	0.5048(4)
θ_W_ (K)	–102.9(3)	–94.2(1)	–113.4(1)	–124.1(2)	–102.0(2)
μ_eff_ (μ_B_ per Cu^2+^)	2.003(9)	1.914(2)	2.037(1)	2.088(3)	2.009(2)
*J*_leg_ (K)	85.35(4)	92.0(4)	98.8(2)	102.8(1)	102(1)
*J*_rung_/*J*_leg_	1.0483(6)	0.816(9)	0.546(6)	0.278(4)	0.11(14)
*g*	2.2234(9)	2.186(2)	2.190(1)	2.1360(5)	2.08(2)

A spin glass state might be an expected ground state of Ba_2_CuTe_1–*x*_W_*x*_O_6_ given the full Te/W disorder on the B″(c) site. We did not observe any ZFC/FC divergence in the dc magnetic susceptibility for any of the samples. We further investigated the possibility of a spin glass state by measuring the ac magnetic susceptibility of the *x* = 0.1 and *x* = 0.3 samples, shown in panels c and d of Figure 5, respectively. The *χ′*_ac_ versus *T* data for each of these samples show no frequency-dependent shift in the curve between 2 and 100 K. Furthermore, no peaks were detected in the imaginary component (*χ″*_ac_ vs *T*) of the ac susceptibility. As such, we find no evidence supporting a spin glass state in Ba_2_CuTe_1–*x*_W_*x*_O_6_.

The dc magnetic susceptibility data between 200 and 400 K were fitted using the Curie–Weiss law as illustrated for Ba_2_CuTe_0.9_W_0.1_O_6_ in Figure 5b. The Curie constants (*C*) and Weiss temperatures (θ_W_) for each sample are listed in Table 2. Generally, there is little change in θ_W_ across the series, implying the interaction strength remains constant. The value of *C* was used to calculate the effective magnetic moment μ_eff_. The value of μ_eff_ is ∼2 μ_B_ per Cu^2+^ for each sample, larger than expected for a Cu^2+^ moment, but close to the previously reported value of 1.96 μ_B_ per Cu^2+^ for Cu^2+^ in Ba_2_CuTeO_6_.^38^ It is unclear why the effective magnetic moment is enhanced in these samples.

**Figure 6:**
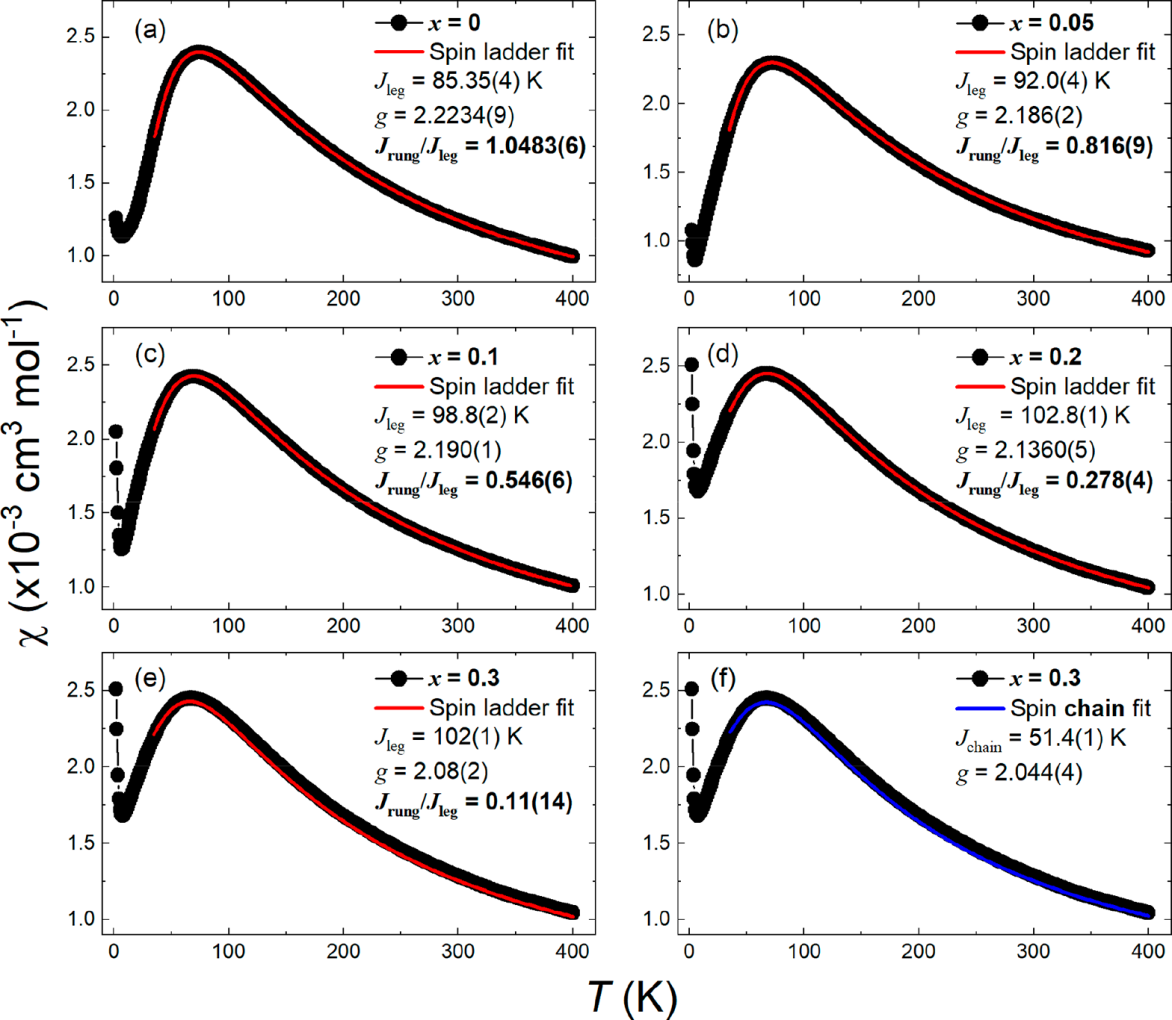
(a–e) Isolated two-leg spin ladder model fits to the Ba_2_CuTe_1–*x*_W_*x*_O_6_ (*x* = 0–0.3) susceptibility data. The spin ladder fit (red line) was performed between 35 and 400 K. The legend contains the fitting parameters *J*_leg_, *J*_rung_/*J*_leg_, and *g*. The strength of the *J*_leg_ interaction is largely unchanged, while the *J*_rung_/*J*_leg_ ratio of the intraladder interactions decreases with *x*. (f) Spin chain model fit (blue line) to the *x* = 0.3 susceptibility data between 35 and 400 K. The spin chain model provides a good description of the χ vs *T* curve at high values of *x*, further supporting the lower *J*_rung_/*J*_leg_ ratio.

The χ versus *T* data were fitted using an isolated two-leg spin ladder model.^52^ This model is based on highly accurate Quantum Monte Carlo (QMC) calculations and allows us to investigate how the intraladder interactions are modified by W^6+^ substitution. The model has three fitting parameters: the main interaction *J*_leg_, the ratio *J*_rung_/*J*_leg_, and *g*. The isolated spin ladder model was previously used to show that intraladder interactions are equally strong in Ba_2_CuTeO_6_ (*J*_rung_/*J*_leg_ ∼ 1),^38,41^ which was in excellent agreement with density functional theory calculations^38^ and subsequent inelastic neutron scattering measurements.^39^Figure 6 shows the magnetic susceptibility of all samples is described well by the isolated spin ladder model with the fitted parameters listed in Table 2. Our fit results for *x* = 0 are in excellent agreement with previous literature.^38,41^ Upon W^6+^ substitution, the strength of the main *J*_leg_ interaction is relatively stable showing a small increase. However, the relative strength of the intraladder interactions changes significantly as *x* increases. The *J*_rung_/*J*_leg_ ratio decreases from near unity (i.e., *J*_leg_ and *J*_rung_ of equal magnitude) for *x* = 0, to a value of *J*_rung_/*J*_leg_ = 0.11(14) upon reaching *x* = 0.3. This shows that W^6+^ doping leads to a strong suppression of the *J*_rung_ interaction.

In terms of the overall strength of the magnetic interactions, *J*_leg_ has twice the impact of *J*_rung_, because *J*_leg_ connects any Cu^2+^ site to two neighboring sites while *J*_rung_ connects to only one. Thus, the effect of the small increase in *J*_leg_ and the strong suppression of *J*_rung_ is a moderate weakening of the overall interactions. This is consistent with the shift in *T*_max_ to lower temperatures, but not with our relatively constant trend in θ_W_ obtained from Curie–Weiss fits. The reason for this discrepancy is not known, but it could be related to the strong quantum fluctuations arising from the nearby quantum critical point in Ba_2_CuTeO_6_.^39^

**Figure 7:**
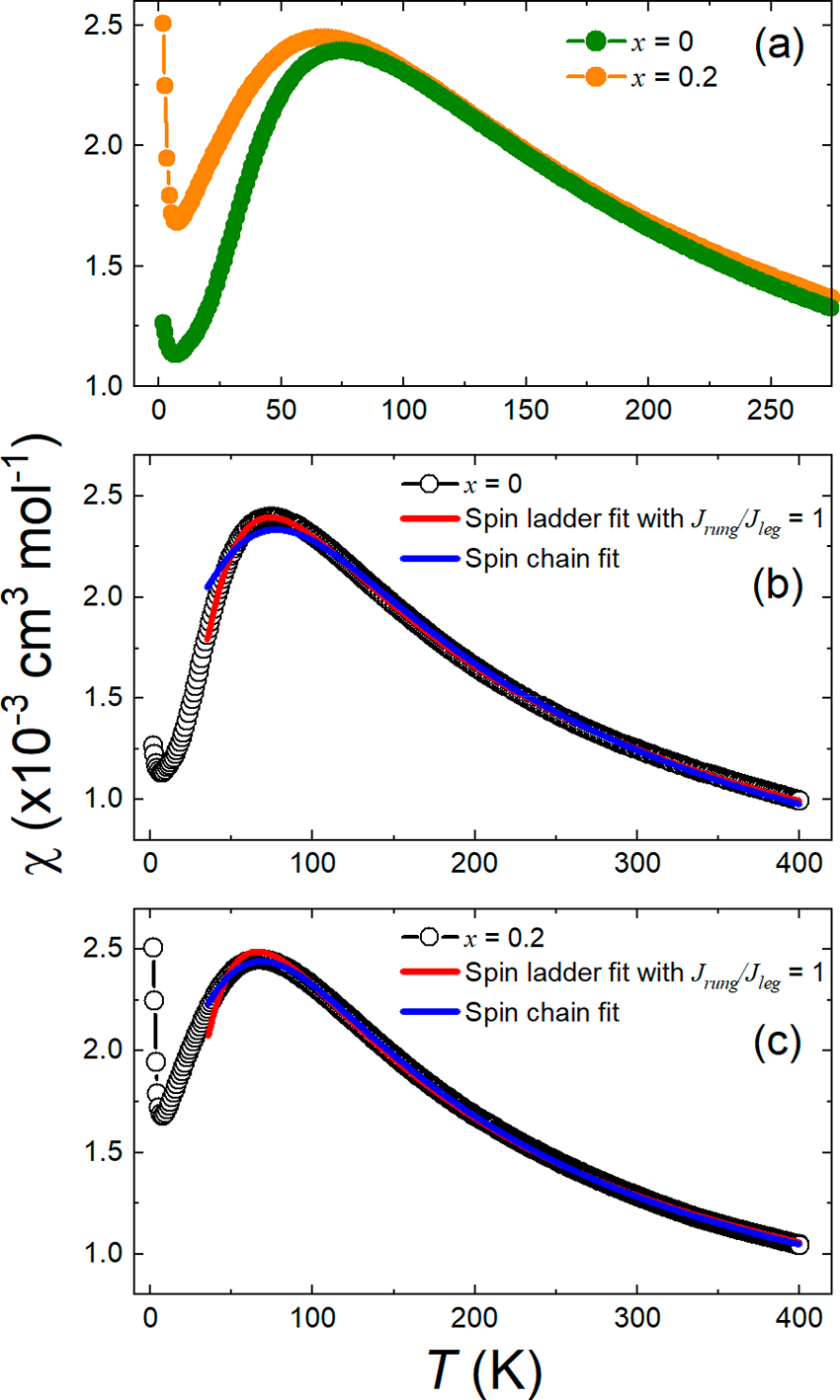
(a) Comparison of the susceptibility curves of *x* = 0 and *x* = 0.2 compounds. The maximum in susceptibility is more symmetric and broader for *x* = 0.2 than for *x* = 0, which is consistent with a lower *J*_rung_/*J*_leg_ ratio. In panels b and c, we compare the isotropic spin ladder (with the *J*_rung_/*J*_leg_ ratio fixed to 1) and spin chain model fitting to the susceptibility data for *x* = 0 and *x* = 0.2, respectively. The spin ladder fits with *J*_rung_/*J*_leg_ = 1 are colored red, and the spin chain fits are colored blue. The isotropic spin ladder model fits the *x* = 0 data very well, but not the *x* = 0.2 data. The susceptibility of *x* = 0.2 is better described with a spin chain model confirming that W^6+^ doping leads to a decrease in the *J*_rung_/*J*_leg_ ratio.

The differences in magnetic susceptibility between an isotropic spin ladder with equally strong *J*_leg_ and *J*_rung_ interactions and more spin chain-type systems with suppressed *J*_rung_ are not immediately obvious. The isolated spin ladder fitting function used here is based on QMC calculations of the magnetic susceptibility for different *J*_rung_/*J*_leg_ ratios.^52^ The QMC calculations show that differences in the high-temperature susceptibility are minor between an isotropic *J*_rung_/*J*_leg_ = 1 spin ladder and a spin chain with the latter showing a small increase. The main difference between the two models is found in the broad maximum at *T*_max_. For isotropic *J*_rung_/*J*_leg_ = 1 ladders, this susceptibility is relatively sharp and highly asymmetric. As the *J*_rung_/*J*_leg_ ratio decreases, the maximum becomes both broader and much more symmetric, while shifting to lower temperatures. We observe these expected trends in our Ba_2_CuTe_1–*x*_W_*x*_O_6_ samples: the maximum becomes broader and more symmetric with an increase in *x* while also shifting to lower temperatures. This further shows that the *J*_rung_/*J*_leg_ ratio does decrease with W^6+^ doping. The differences in the broad maximum are highlighted in Figure 7a, which shows a comparison of the magnetic susceptibilities of *x* = 0 and *x* = 0.2. It should be noted that the Curie-like feature observed at low temperatures is too small to explain the changes in the broad maximum.

To conclusively show that *J*_rung_/*J*_leg_ decreases with an increase in *x* in Ba_2_CuTe_1–*x*_W_*x*_O_6_, we compare the best fits from an isotropic spin ladder model^52^ (*J*_rung_/*J*_leg_ = 1) and a spin chain model^53,54^ (*J*_rung_/*J*_leg_ = 0) for *x* = 0 and *x* = 0.2 compounds in panels b and c of Figure 7. The isotropic spin ladder model fits *x* = 0 very well, and the *J*_rung_/*J*_leg_ ratio of ∼1 has been confirmed by density functional theory calculation and inelastic neutron scattering.^38,39^ As expected for the *x* = 0 compound, the spin chain model provides a poor fit for the maximum in susceptibility, but also for the high-temperature susceptibility. In contrast, the isotropic spin ladder model provides a poor fit for the *x* = 0.2 data, especially for the broad maximum. The spin chain model, however, describes the *x* = 0.2 data well and provides a noticeably better fit than the isotropic spin ladder model for the maximum but also at high temperatures. This confirms W^6+^ doping in Ba_2_CuTe_1–*x*_W_*x*_O_6_ changes the relative strength of magnetic interactions by decreasing the *J*_rung_/*J*_leg_ ratio. Spin chain fits to all compounds are presented in Figure S29, and this model provides a progressively better fit to susceptibility data with an increase in *x*.

#### 3.2.2 Heat Capacity

**Figure 8:**
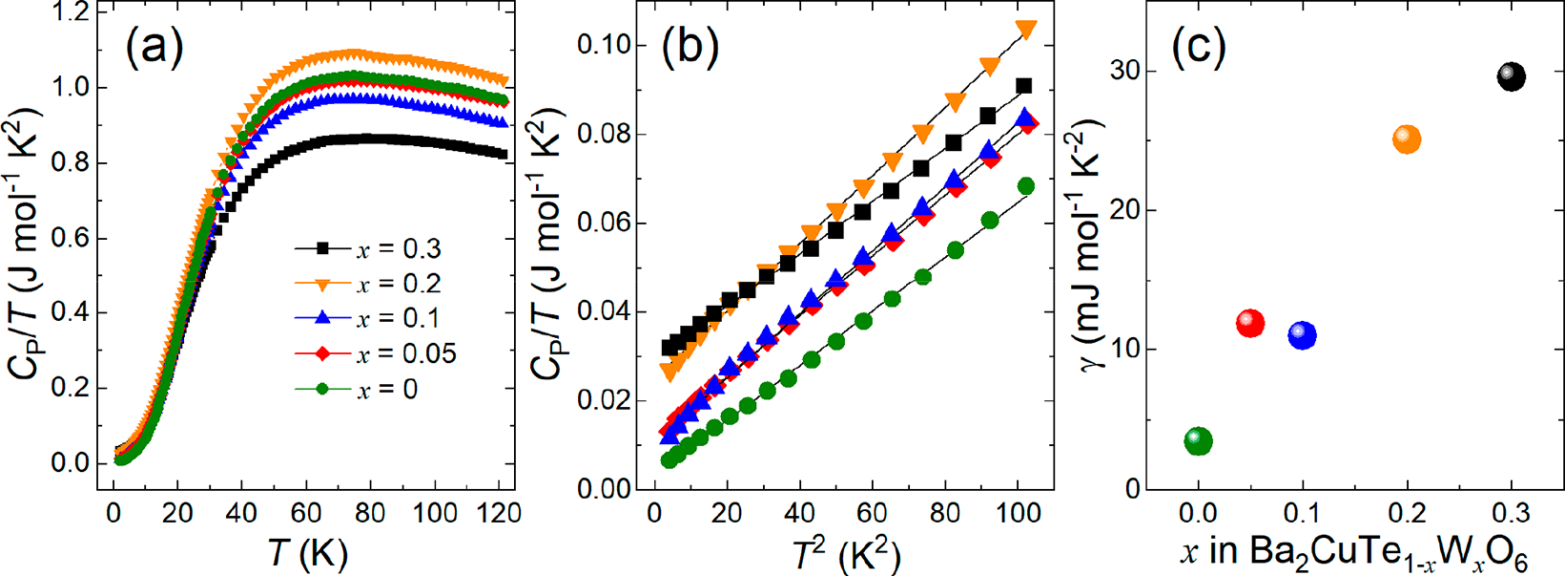
Heat capacity data for Ba_2_CuTe_1–*x*_W_*x*_O_6_ (*x* = 0–0.3), including (a) *C_P_*/*T* vs *T* curves for all samples, (b) Debye–Einstein fits of *C_P_*/*T* vs *T*^2^ data between 2 and 10 K, and (c) electronic γ term contribution to *C_P_* as a function of *x* in Ba_2_CuTe_1–*x*_W_*x*_O_6_.

Zero-field heat capacity (*C_P_*) measurements were performed on all samples. The plot in Figure 8a shows the *C_P_*/*T* data as a function of *T*. Close examination of the curves shows no evidence of an ordering transition in the parent or doped samples. The former observation agrees with previous heat capacity measurements on Ba_2_CuTeO_6_.^38,40^ Because of weak Néel ordering, Ba_2_CuTeO_6_ possesses strong quantum fluctuations that spread out the magnetic entropy. Hence, *C_P_*/*T* measurements are largely insensitive to any trace of a λ ordering peak about *T*_N_. The lack of a λ peak in the *x* > 0 curves shows magnetism in the doped samples is similarly weak, as expected from the small *S* = ^1^/_2_ Cu^2+^ moment and low-dimensional behavior. Consequently, the curves appear to be much the same, so it is not possible to distinguish differences in magnetic ordering. The small variation in the high-temperature data is an artifact of the silver contribution to *C_P_*/*T*, which is very well corrected for at low temperatures. However, there are notable trends in the low-temperature *C_P_*/*T* data. The expansion of the range of 2–10 K in Figure 8b shows a linear relationship between *C_P_*/*T* and *T*^2^ that is readily fitted using the Debye–Einstein model (*C_P_* = *γT* + β_D_*T*^3^). The electronic contribution (γ) to the heat capacity was extracted from the intercept of *C_P_*/*T* versus *T*^2^ and plotted as a function of *x* in Figure 8c. For the *x* = 0 sample, the value of γ is almost zero, in excellent agreement with previous studies.^38^ However, as *x* increases, so does the electronic contribution to *C_P_*, until γ reaches 29.6 mJ mol^–1^ K^–2^ for the *x* = 0.3 sample. Given these materials are Mott insulators, this electronic contribution can be associated with only magnetic excitations, not conduction electrons.

## 4 Discussion

**Figure 9:**
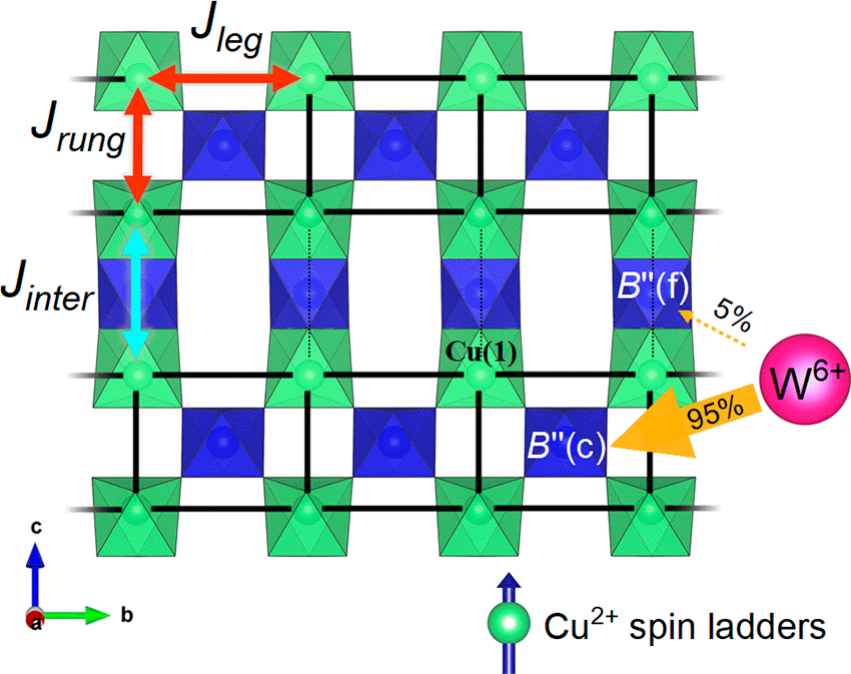
Diagram illustrating the strong W^6+^ preference for the corner-sharing B″(c) site vs the face-sharing B″(f) site in the Ba_2_CuTe_1–*x*_W_*x*_O_6_ structure. The spin ladder structure shown is the same in the *C*2/*m* and *P*1 phases. The solid black lines represent the intraladder interactions *J*_leg_ and *J*_rung_ (red arrows) of the Cu^2+^ spin ladder structure. The dotted lines represent the main *J*_inter_ interladder interaction (blue arrow) between the spin ladders. The strong B″(c) site preference means the intraladder interactions are most affected by the W^6+^ d^0^ orbitals.

Our in-depth structural analysis using a combination of synchrotron X-ray diffraction, neutron diffraction, and EXAFS shows W^6+^ is site-selectively doped onto the corner-sharing B″(c) site. As illustrated in Figure 9, this means the intraladder, *J*_rung_ and *J*_leg_, interactions are most affected by W^6+^ doping of Ba_2_CuTeO_6_. The minor occupation (<5%) of the B″(f) site is unlikely to have a significant effect on the interladder interactions. Hence, d^10^/d^0^ doping on the B″(c) site directly tunes the intraladder Cu–O–B″(c)–O–Cu superexchange, while the interladder Cu–O–B″(f)–O–Cu exchange remains unchanged (Figure 9).

W^6+^ doping has only a weak effect on the structure of Ba_2_CuTe_1–*x*_W_*x*_O_6_, as expected from the similar ionic radii of W^6+^ (0.6 Å) and Te^6+^ (0.56 Å).^55^ The hexagonal layered structure and, hence, the Cu^2+^ spin ladder geometry remained intact across the solid solution. There are only a few minor differences. Mainly, the variable-temperature neutron diffraction data show W^6+^ doping reduced the *C*2/*m* to *P*1 structural transition temperature from just below room temperature (*x* = 0) to ∼100–120 K (*x* = 0.3). The *C*2/*m* to *P*1 transition is weak; therefore, the spin ladder structure and magnetic interactions remain the same.

The synchrotron X-ray data revealed the strong selectivity for W^6+^ to reside on the corner-sharing B″(c) site. Across the Ba_2_CuTe_1–*x*_W_*x*_O_6_ series, the structural model provided the best fit to the data when the majority (∼95%) of the W^6+^ dopant present in the sample resided on the B″(C) site. The EXAFS data corroborated this result, reproducing the experimental data only when the model placed W^6+^ exclusively on the B″(c) site. The strong site selectivity appears to be surprising for a few reasons, first due to the aforementioned nearly identical W^6+^ and Te^6+^ ionic radii.^56^ This, and the identical +6 charge, lends us to expect a random distribution of Te^6+^ and W^6+^ across the B″(c) and B″(f) sites. However, this argument neglects consideration of the metal–oxygen bonding differences in Te^6+^ and W^6+^ (as well as Mo^6+^) perovskite structures.

It has been noted that perovskites containing W^6+^ and Mo^6+^ exclusively form double perovskite structures, whereas Te^6+^-containing perovskites can also adopt hexagonal structures.^57^ The W^6+^ and Mo^6+^ cations inability to form hexagonal structures stems from the differences in metal–oxygen bonding involving d^0^ versus d^10^ cations. In the case of Te^6+^, the filled 4d^10^ orbitals limit the d-orbital contribution to metal–oxygen bonding, creating a significant s- and p-orbital contribution in Te–O bonding. This directs the electron density toward the oxide anions and away from the octahedral surface, thus helping to weaken cation–cation repulsion between Te^6+^ and the surrounding B′ cation.^57^ The weakened cation–cation repulsion allows Te^6+^ to occupy face-sharing sites in hexagonal perovskite structures, such as the B″(f) site in Ba_2_CuTe_1–*x*_W_*x*_O_6_. The opposite is true for W^6+^ and Mo^6+^ perovskites where the 5d^0^ orbitals do contribute significantly to metal–oxygen bonding, leading to a strong π-orbital contribution. These π-bonding interactions generate highly regular [WO_6_]^6–^ octahedral units, with a more spherical charge distribution.^58^ This produces a relatively strong repulsion across shared octahedral faces, making face-sharing unfavorable. Consequently, W^6+^ and Mo^6+^ prefer corner-sharing sites where cations are farther apart and therefore do not form hexagonal structures.

This electrostatic energetic penalty explains why W^6+^ strongly prefers the corner-sharing B″(c) site, where the distance to the Cu^2+^ cation is significantly larger than that of the face-sharing B″(f) site in the Cu–B″(f)–Cu trimer, e.g., Te^6+^–Cu^2+^ distances of 3.962(2) Å (face-sharing) and 2.738(1) Å (face-sharing) in *x* = 0.3 at 300 K. The unfavorability of the B″(f) site also explains why attempts to synthesize richer W^6+^ compositions beyond *x* = 0.3 failed. Furthermore, this reasoning is also the basis for why Ba_2_CuTeO_6_ and Ba_2_CuWO_6_ adopt different structures. The difference in metal–oxygen bonding drives W^6+^ to form tetragonal Ba_2_CuWO_6_ to maximize the cation distances, while Te^6+^ can accommodate face-sharing in hexagonal Ba_2_CuTeO_6_.^57^ Note that while seemingly stronger covalency in Te–O may imply stronger superexchange interactions, this is not necessarily the case. Superexchange between corner-sharing CuO_6_ and TeO_6_ octahedra mainly occurs via a Cu–O–O–Cu pathway without a significant contribution from the Te^6+^ 4d^10^ states, which lie far below the Fermi level, or the Te^6+^ 5s and 5p states.^12,13^ In contrast, the empty W^6+^ 5d^0^ orbitals hybridize strongly with O 2p, resulting in significant Cu–O–W–O–Cu superexchange. This results in different dominant interactions for Te^6+^ and W^6+^ compounds, although the prediction of the overall strength of magnetic interactions remains difficult due to competing effects.

The d^0^ W^6+^ cations were site-selectively doped onto the B″(c) site, which connects the intraladder *J*_leg_ and *J*_rung_ interactions via Cu–O–B″(c)–O–Cu superexchange. Therefore, one would expect the d^10^/d^0^ doping to result in the direct tuning of these intraladder interactions in Ba_2_CuTe_1–*x*_W_*x*_O_6_. This does in fact happen as confirmed by our isolated spin ladder fits to magnetic susceptibility data. Tungsten doping has a significant effect on the relative strengths of the intraladder interactions: the *J*_rung_/*J*_leg_ ratio decreased from ∼1 for the *x* = 0 sample as the W^6+^ content increased, reaching a *J*_rung_/*J*_leg_ value of ∼0.1 for the *x* = 0.3 sample. *J*_leg_ was approximately constant with an increase in *x*, therefore showing decreases in *J*_rung_/*J*_leg_ originate from a weakening of the *J*_rung_ interaction. Such a modification of the relative intraladder strength by W^6+^ means that as *J*_rung_/*J*_leg_ approaches zero, the system is progressively tuned from a spin ladder toward an isolated spin chain.

While our results show that W^6+^ doping has a significant effect on the magnetic interactions in Ba_2_CuTe_1–*x*_W_*x*_O_6_, the true magnetic ground states of the doped samples remain unknown. We are unable to rule out the presence of magnetic order in the doped samples despite the lack of magnetic Bragg peaks in the neutron diffraction patterns. This is because magnetic scattering from Cu^2+^*S* = ^1^/_2_ moments is very weak and HRPD is an instrument optimized for structure solution as opposed to magnetism.

Magnetic susceptibility and heat capacity data provide hints that the magnetic ground state of Ba_2_CuTe_1–*x*_W_*x*_O_6_ might change upon doping. We observe an increasing Curie tail in the dc susceptibility with an increase in the level of d^10^/d^0^ doping. More importantly, a significant *T*-linear γ term is observed in the heat capacity data for doped samples, but not for pure Ba_2_CuTeO_6_. This large γ term has no obvious origin in a magnetically ordered insulator. The high degree of Te/W disorder on the B″(c) site along with magnetic frustration could lead to a spin glass state, which would explain the γ term in the heat capacity.^59^ However, our ac susceptibility measurements show no evidence of a spin glass state down to 2 K. Another possibility is that a quantum disordered state, such as a random singlet state, might form in Ba_2_CuTe_1–*x*_W_*x*_O_6_ as it forms in Sr_2_CuTe_1–*x*_W_*x*_O_6_. This would explain both the Curie-tail feature in the magnetic susceptibility and the γ term in the heat capacity.^60,61^ For the *x* = 0.3 sample, the γ term approaches 50% of the value for Sr_2_CuTe_0.5_W_0.5_O_6_.^8^ The ground state of the doped samples could be further investigated using muon spin rotation and relaxation and additional neutron scattering experiments.

## 5 Conclusions

Chemical doping of the hexagonal perovskite Ba_2_CuTeO_6_ delivers a Ba_2_CuTe_1–*x*_W_*x*_O_6_ solid solution (0 ≤ *x* ≤ 0.3). Structural differences among the *x* = 0, 0.05, 0.1, 0.2, and 0.3 samples were investigated using a combination of neutron diffraction, synchrotron X-ray diffraction, and EXAFS. This revealed a strong site selectivity for W^6+^ cations to occupy the corner-sharing B″(c) site within the intraladder structure. The site selectivity results from differences in molecular bonding that leads W^6+^ to prefer the corner-sharing site. Site-specific d^10^/d^0^ doping directly modifies the intraladder interactions by suppressing *J*_rung_ as the level of W^6+^ doping increases, while *J*_leg_ remains constant. While it is unclear what type of ground state this creates, it is clear the direct d^10^/d^0^ effect has a significant effect on the magnetic interactions. As the level of W^6+^ doping increases, *J*_rung_ is further suppressed, and the system is tuned from a spin ladder toward a spin chain as *J*_rung_/*J*_leg_ approaches zero.

Overall, this work demonstrates that the d^10^/d^0^ effect can be extended beyond double perovskite structures to modify the magnetic interactions in hexagonal perovskites. Furthermore, the effect could be extended to any structural type that contains corner-sharing octahedra, such as perovskite-derived two-dimensional structures (e.g., Ruddlesden–Popper phases and Dion–Jacobson phases). This could even be done in a site-selective manner as we have shown here for Ba_2_CuTe_1–*x*_W_*x*_O_6_. Therefore, our work highlights the d^10^/d^0^ effect as a powerful tool for tuning magnetic interactions and ground states in perovskite-derived oxides.

## Supporting Information Available

Laboratory X-ray diffraction pattern, Rietveld results from neutron diffraction data, including bond lengths and angles, Rietveld refinement of synchrotron X-ray diffraction data, analysis of EXAFS data, and Curie–Weiss fitting of the magnetic susceptibility data (PDF)

## Accession Codes

CCDC 2128596–2128600 contain the supplementary crystallographic data for this paper. These data can be obtained free of charge via www.ccdc.cam.ac.uk/data_request/cif, or by emailing data_request@ccdc.cam.ac.uk, or by contacting The Cambridge Crystallographic Data Centre, 12 Union Road, Cambridge CB2 1EZ, UK; fax: +44 1223 336033.
