# Supplementary material for: Site-Selective d10/d0 Substitution in an S = 1/2 Spin Ladder Ba2CuTe1–xWxO6 (0 ≤ x ≤ 0.3)
Source: Inorg Chem. 2022 Feb 21;61(9):4033–45. doi: 10.1021/acs.inorgchem.1c03655 (PMC9007447; doi:10.1021/acs.inorgchem.1c03655)
Supplement: Supplementary file 1 — ic1c03655_si_001.pdf [file ic1c03655_si_001.pdf]

Supporting information

Site-selective d^{10}/d^0 substitution in an S = ½ spin ladder

$Ba_2CuTe_{1-x}W_xO_6$ ($0 \le x \le 0.3$)

Charlotte Pughe[1], Otto H. J. Mustonen[1,2], Alexandra S. Gibbs[3,4,5], Martin Etter[6], Cheng Liu[7], Siân E. Dutton[7], Aidan Friskney[1], Neil C. Hyatt[1], Gavin B. G. Stenning[4], Heather M. Mutch[1], Fiona C. Coomer[8], Edmund J. Cussen[1]**

1. Department of Material Science and Engineering, University of Sheffield, Sheffield S1 3JD, United Kingdom

2. School of Chemistry, University of Birmingham, Edgbaston, Birmingham B15 2TT, United Kingdom

3. School of Chemistry, University of St Andrews, North Haugh, St Andrews, KY16 9ST

4. ISIS Pulsed Neutron and Muon Source, STFC Rutherford Appleton Laboratory, Didcot OX11 0QX, United Kingdom

5. Max Planck Institute for Solid State Research, Heisenbergstrasse 1, 70569 Stuttgart

6. Deutsches Elektronen-Synchrotron (DESY), 22607 Hamburg, Germany

7. Cavendish Laboratory, University of Cambridge, J.J. Thomson Avenue, Cambridge, CB3 0HE, United Kingdom

8. Johnson Matthey Battery Materials, Reading RG4 9NH, United Kingdom

Corresponding Authors (*)

Edmund J. Cussen. e.j.cussen@sheffield.ac.uk

Otto H. J. Mustonen. ohj.mustonen@gmail.com

1. Laboratory X-ray diffraction (Rigaku Minflex)

Figure S1 shows an example laboratory X-ray diffraction pattern of $Ba_2CuTe_{0.9}W_{0.1}O_6$ collected using a Rigaku Miniflex diffractometer (300 K, Cu $K\alpha_1$/ $K\alpha_2$ (λ = 1.5405 and 1.5443 Å). The inset shows the unit cell volume change across the solid solution between x = 0 to 0.3.

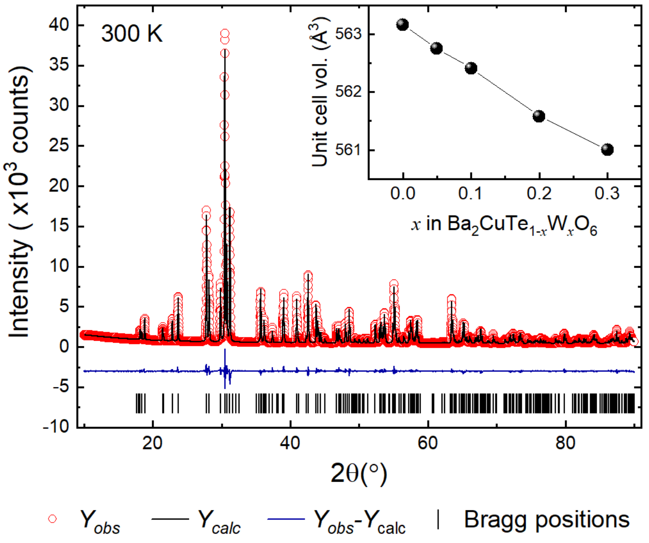

Figure S1. Laboratory X-ray diffraction pattern of $Ba_2CuTe_{0.9}W_{0.1}O_6$. The inset shows the unit cell volume change as a function of x in $Ba_2CuTe_{1-x}W_xO_6$. The error bars for the unit cell volumes are smaller than the data points.

2. Neutron diffraction

Neutron diffraction data were processed using the following method. The raw data from all three banks were normalized against the vanadium standard and experimentally corrected for sample absorption. The three banks were then simultaneously refined using the Rietveld method in GSAS-2 with either the monoclinic ($C2/m$) or triclinic ($P\bar{1}$) models.[1] The background was modelled using the Chebyshev function with 10 points. The unit cell volume, atomic positions and Uiso values were refined with the Uiso values for Te and W on the B''(c) and B''(f) sites constrained to be equivalent. The refined structural models used the refined B''(c) and B''(f) site fractions for x = 0.1 and x = 0.3 determined from the synchrotron X-ray diffraction results.

In addition to the above, DIFFA was refined for all banks. Refining additional profile parameters such as DIFFC (lower resolution banks 2 and 3 only) and sigma-1 for all banks, led to a very marginal improvement in the fit. Hence, these parameters were not refined. It was necessary to include sample strain which was modelled using the generalized model. Positive Uiso values were obtained at all temperatures throughout the monoclinic phase. However, during the test refinements within the triclinic phase, the Ba(1) site Uiso became negative. This is because as the temperature is reduced, the Ba(1) site Uiso value becomes very close to zero. Therefore, in the final refinements the Ba(1) and Ba(2) sites Uiso values were constrained to be equivalent, producing a stable refinement with positive Uiso values for all atoms. Constraining the Ba(1) and Ba(2) sites has negligible effect on the fit quality. The tables below show the refined structures of $Ba_2CuTe_{0.9}W_{0.1}O_6$ and $Ba_2CuTe_{0.7}W_{0.3}O_6$ at ~2 K and 300 K.

Table S1. Refined structure of $Ba_2CuTe_{0.9}W_{0.1}O_6$ at 1.55 K

Space Group: $P\bar{1}$, No. 2, 1.44 K<br>R_P = 3.45%, R_{wp} = 3.97%, R_{exp} = 1.66%, χ^2 = 8.94, 111 var.<br>a = 5.7065(2) Å, b = 5.8490(2) Å, c = 10.2555(7) Å<br>α = 108.464(1)°, β = 107.0747(8)°, γ = 60.7373(3)°<br>*Vol.* = 279.233(5) Å^3						
Site	Wyckoff Position	*x*	*y*	*z*	Site fraction	Uiso (Å^2)
Ba1	2i	0.13093(40)	0.12213(35)	0.38101(19)	1.0	0.00192(32)
Ba2	2i	0.27722(40)	0.28124(34)	0.85036(18)	1.0	0.0019
Te1	1a	0	0	0	0.809	0.00403(53)
W1	1a	0	0	0	0.191	0.00403
Te2	1h	0.5	0.5	0.5	0.991	0.00388(56)
W2	1h	0.5	0.5	0.5	0.009	0.00388(56)
Cu1	2i	0.41345(26)	0.40522(25)	0.21466(12)	1.0	0.0055(38)
O1	2i	0.62858(35)	0.58280(40)	0.37455(17)	1.0	0.00619(47)
O2	2i	0.16934(33)	0.57361(38)	0.36287(19)	1.0	0.00551(45)
O3	2i	0.35992(37)	0.86776(31)	0.59887(18)	1.0	0.00793(48)
O4	2i	0.79881(42)	0.78944(41)	0.90323(18)	1.0	0.00710(44)
O5	2i	0.26919(41)	0.78131(40)	0.87853(19)	1.0	0.00631(49)
O6	2i	0.20133(42)	0.76452(33)	0.12686(18)	1.0	0.00790(48)

Table S2. Refined structure of $Ba_2CuTe_{0.9}W_{0.1}O_6$ at 300 K

Space Group: $C2/m$, No. 12, 300 K<br>R_P = 3.10%, R_{wp} = 3.85%, R_{exp} = 1.22%, χ^2 = 19.10, var. 86<br>a = 10.2278(2) Å, b = 5.72160(4) Å, c = 10.0958(2) Å, β = 107.9556(5)°<br>*Vol.* = 562.03(1) Å^3						
Site	Wyckoff Position	*x*	*y*	*z*	Site fraction	Uiso (Å^2)
Ba1	4i	0.12823(18)	0	0.37994(18)	1.0	0.00553(46)
Ba2	4i	0.28256(19)	0	0.84900(18)	1.0	0.01041(50)
Te1	2a	0	0	0	0.809	0.00704(57)
W1	2a	0	0	0	0.191	0.00704(57)
Te2	2d	0	0.5	0.5	0.991	0.00761(56)
W2	2d	0	0.5	0.5	0.009	0.00761(56)
Cu1	4i	-0.09421(13)	0.5	0.21445(11)	1.0	0.00981(41)
O1	4i	0.13295(17)	0.5	0.40048(18)	1.0	0.01445(50)
O2	8j	-0.10503(11)	0.72871(21)	0.36907(11)	1.0	0.01020(36)
O3	4i	0.31753(19)	0.5	0.87535(19)	1.0	0.01884(54)
O4	8j	0.04970(14)	0.76066(27)	0.88914(13)	1.0	0.01698(40)

Table S3. Refined structure of $Ba_2CuTe_{0.7}W_{0.3}O_6$ at 1.44 K

Space Group: $P\bar{1}$, No. 2, 1.44 K<br>R_P = 4.18%, R_{wp} = 4.72%, R_{exp} = 1.75%, χ^2 = 11.97, 111 var.<br>*a* = 5.7008(4) Å, *b* = 5.8402(5) Å, *c* = 10.223(1) Å<br>α = 108.250(2)°, β = 106.693(2)°, γ = 60.7631(6)°<br>*Vol.* = 278.328(9) Å^3						
Site	Wyckoff Position	*x*	*y*	*z*	Site fraction	Uiso (Å^2)
Ba1	2i	0.13036(72)	0.12442(51)	0.38204(29)	1.0	0.00091(45)
Ba2	2i	0.28212(74)	0.28020(49)	0.85115(27)	1.0	0.00091(45)
Te1	1a	0	0	0	0.43	0.00363(78)
W1	1a	0	0	0	0.57	0.00363(78)
Te2	1h	0.5	0.5	0.5	0.97	0.00354(79)
W2	1h	0.5	0.5	0.5	0.03	0.00354(79)
Cu1	2i	0.40939(45)	0.4028(38)	0.21492(18)	1.0	0.00605(54)
O1	2i	0.62719(60)	0.58563(71)	0.37253(29)	1.0	0.00537(76)
O2	2i	0.16580(58)	0.57569(67)	0.36446(31)	1.0	0.00490(73)
O3	2i	0.36352(71)	0.86942(46)	0.59997(28)	1.0	0.01037(70)
O4	2i	0.80705(75)	0.78764(76)	0.89856(34)	1.0	0.00751(74)
O5	2i	0.28312(74)	0.78123(74)	0.88446(36)	1.0	0.00682(79)
O6	2i	0.19415(73)	0.76774(51)	0.12851(27)	1.0	0.00822(73)

Table S4. Refined structure of $Ba_2CuTe_{0.7}W_{0.3}O_6$ at 300 K

Space Group: $C2/m$, No. 12, 300 K<br>R_P = 3.35%, R_{wp} = 3.84%, R_{exp} = 1.74%, χ^2 = 11.02, var. 86<br>*a* = 10.2118(3) Å, *b* = 5.71745(5) Å, *c* = 10.0866(3) Å, β = 107.9193(6)°<br>*Vol.* = 560.35(1) Å^3						
Site	Wyckoff Position	*x*	*y*	*z*	Site fraction	Uiso (Å^2)
Ba1	4i	0.12841(23)	0	0.38015(22)	1.0	0.00394(55)
Ba2	4i	0.28318(25)	0	0.84944(23)	1.0	0.01013(63)
Te1	2a	0	0	0	0.43	0.00850(75)
W1	2a	0	0	0	0.57	0.00850(75)
Te2	2d	0	0.5	0.5	0.97	0.00865(70)
W2	2d	0	0.5	0.5	0.03	0.00865(70)
Cu1	4i	-0.09455(17)	0.5	0.21486(14)	1.0	0.01123(51)
O1	4i	0.13336(23)	0.5	0.39967(23)	1.0	0.01726(64)
O2	8j	-0.10543(14)	0.72904(27)	0.36959(14)	1.0	0.01200(45)
O3	4i	0.31705(24)	0.5	0.87607(23)	1.0	0.01712(65)
O4	8j	0.04946(17)	0.76007(34)	0.88959(16)	1.0	0.01577(48)

a) Monoclinic to triclinic phase transition

The $C2/m$ to $P\bar{1}$ transition temperature (T_{trans}) was determined by plotting the global R_{wp} and χ^2 as a function of temperature (Figure S2 and Figure S3). The lower R_{wp} and high χ^2 at base (1.55 K or 1.44 K), 100 K, 200 K and 300 K reflects the longer counting time used for these datasets. On cooling, the rise in the value of R_{wp} and χ^2 shows the failure of the monoclinic model. Below this temperature, the triclinic model becomes a better description of the structure. T_{trans} occurs between 240-235 K in $Ba_2CuTe_{0.9}W_{0.1}O_6$ and between 100-120 K in $Ba_2CuTe_{0.7}W_{0.3}O_6$. The transition $C2/m$ to $P\bar{1}$ can also be

observed from the evolution of peak splitting with reduced temperature. This is shown in the waterfall plots in Figure S4.

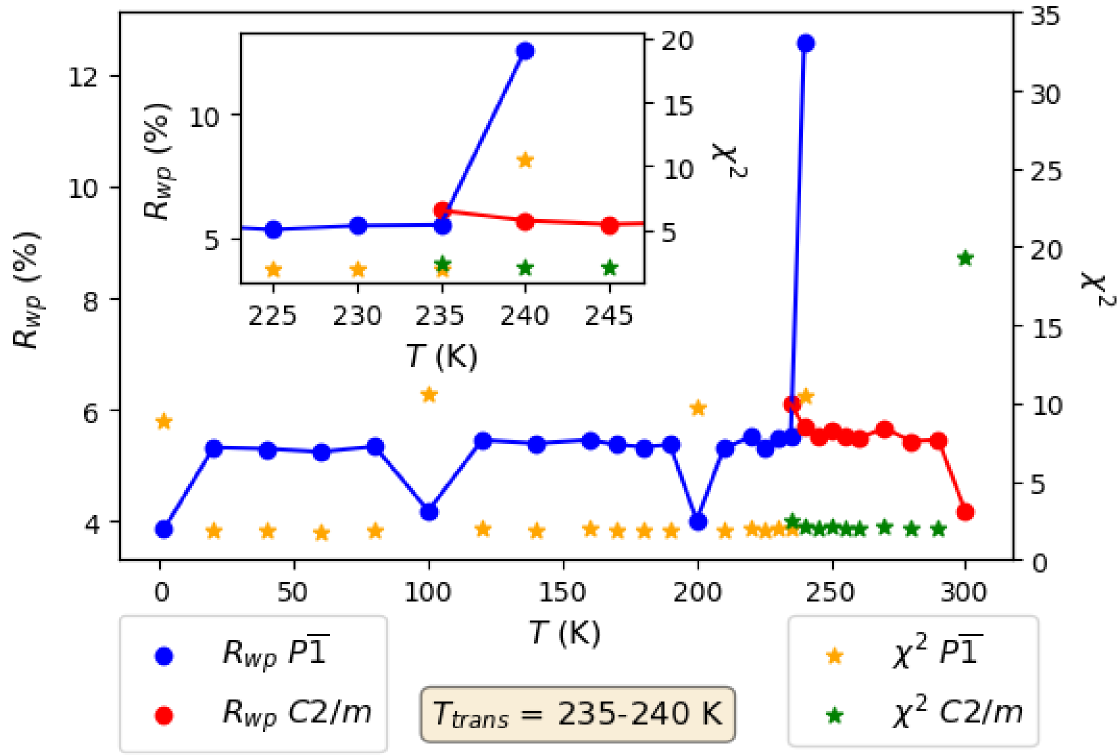

Figure S2. *R*-values vs *T* for $Ba_2CuTe_{0.9}W_{0.1}O_6$ neutron data. The left y-axis shows R_{wp} and the right y-axis shows χ^2 as a function of temperature, *T*.

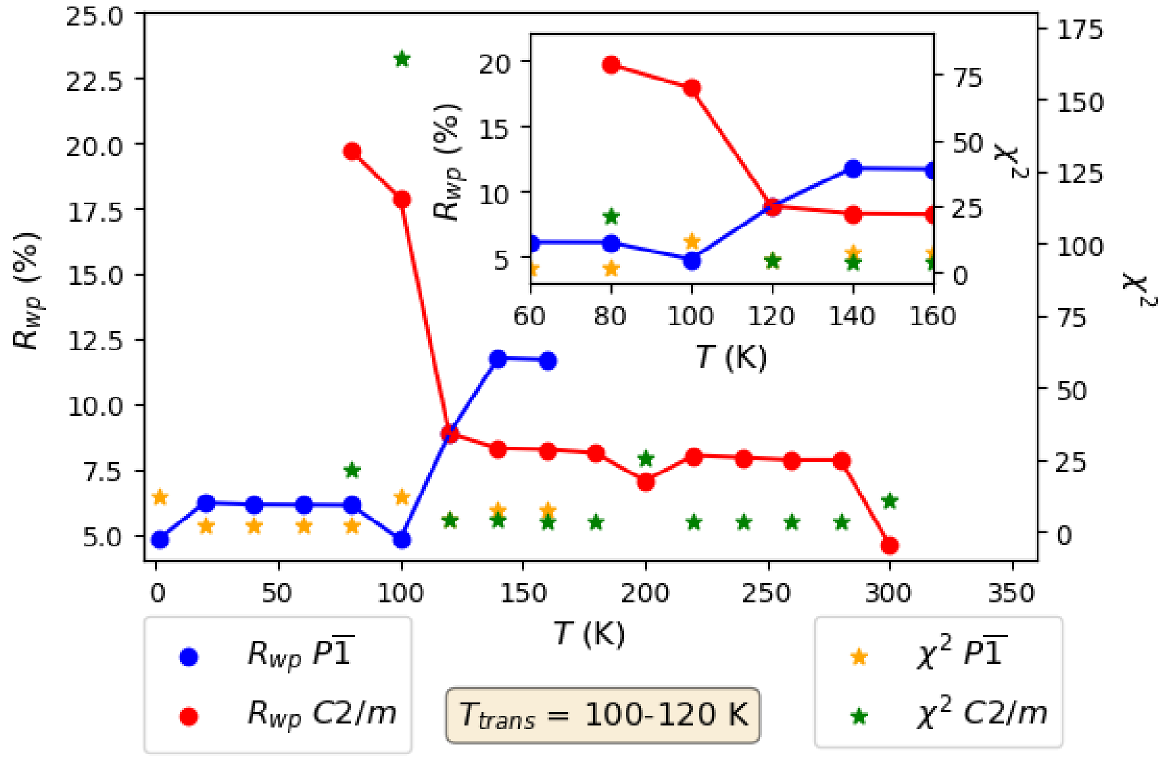

Figure S3. *R*-values vs *T* for $Ba_2CuTe_{0.7}W_{0.3}O_6$ neutron data. The left y-axis shows R_{wp} and the right y-axis shows χ^2 as a function of temperature, *T*.

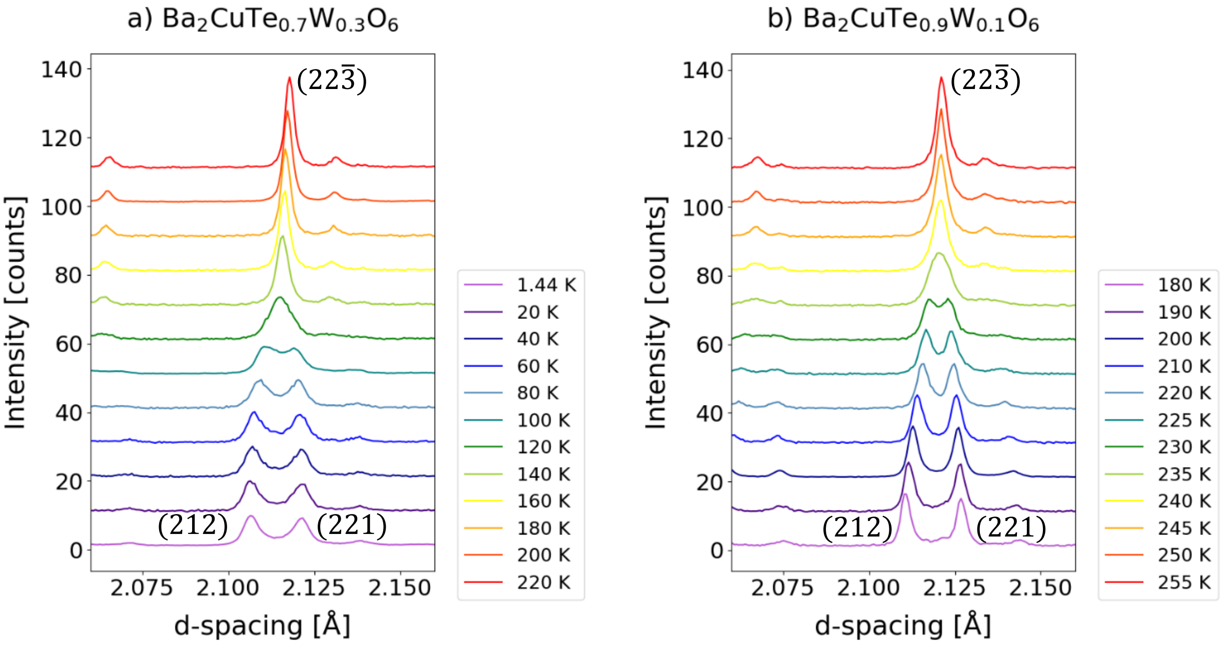

Figure S4. Waterfall plots showing the evolution of peak splitting with reduced temperature in the HRPD neutron diffraction data of (a) $Ba_2CuTe_{0.7}W_{0.3}O_6$ and (b) $Ba_2CuTe_{0.9}W_{0.1}O_6$. The waterfall plots for x = 0.1 and 0.3 show splitting of the $(22\bar{3})$ peak of the $C2/m$ phase into the (212) and (221) peaks of the $P\bar{1}$ phase.

b) Lattice parameters determined from Rietveld analysis of neutron diffraction data.

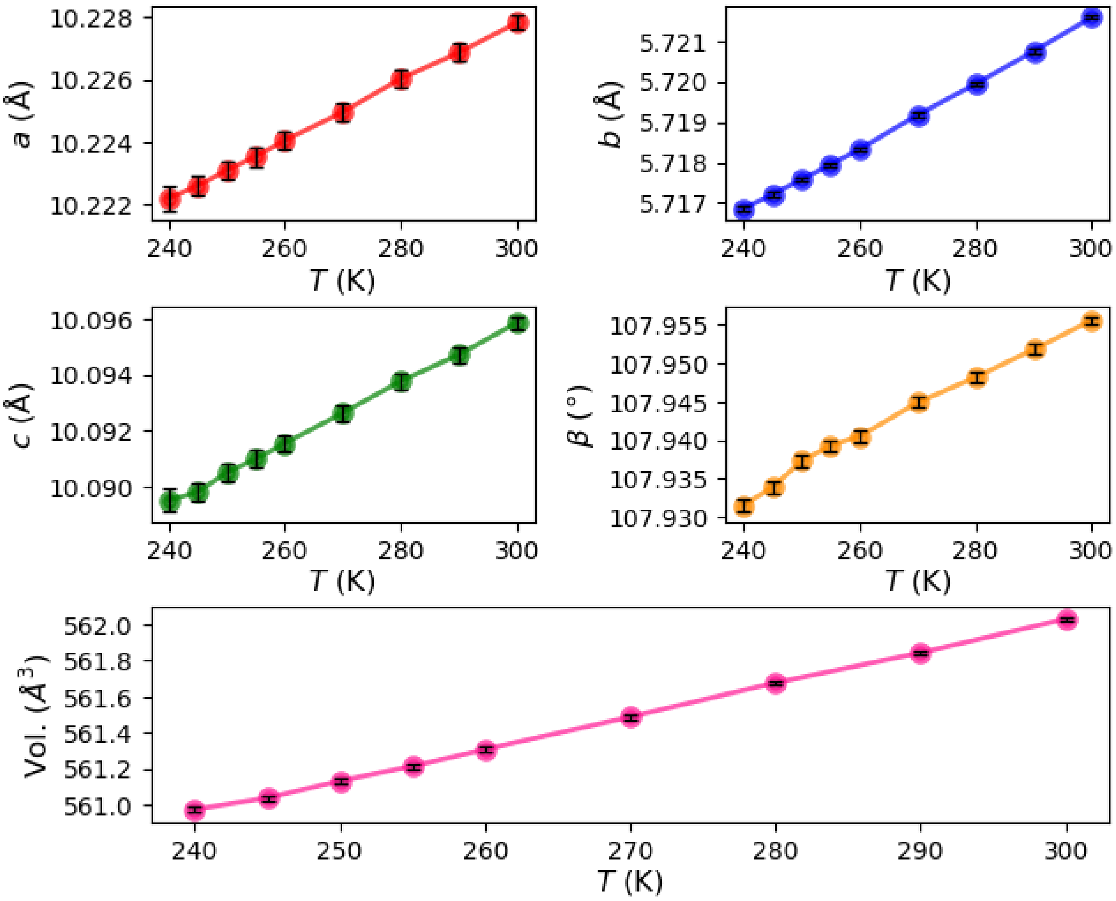

Figure S5. Unit cell parameters and unit cell volume of $Ba_2CuTe_{0.9}W_{0.1}O_6$ in the monoclinic phase, $C2/m$, as a function of *T* between 240-300 K.

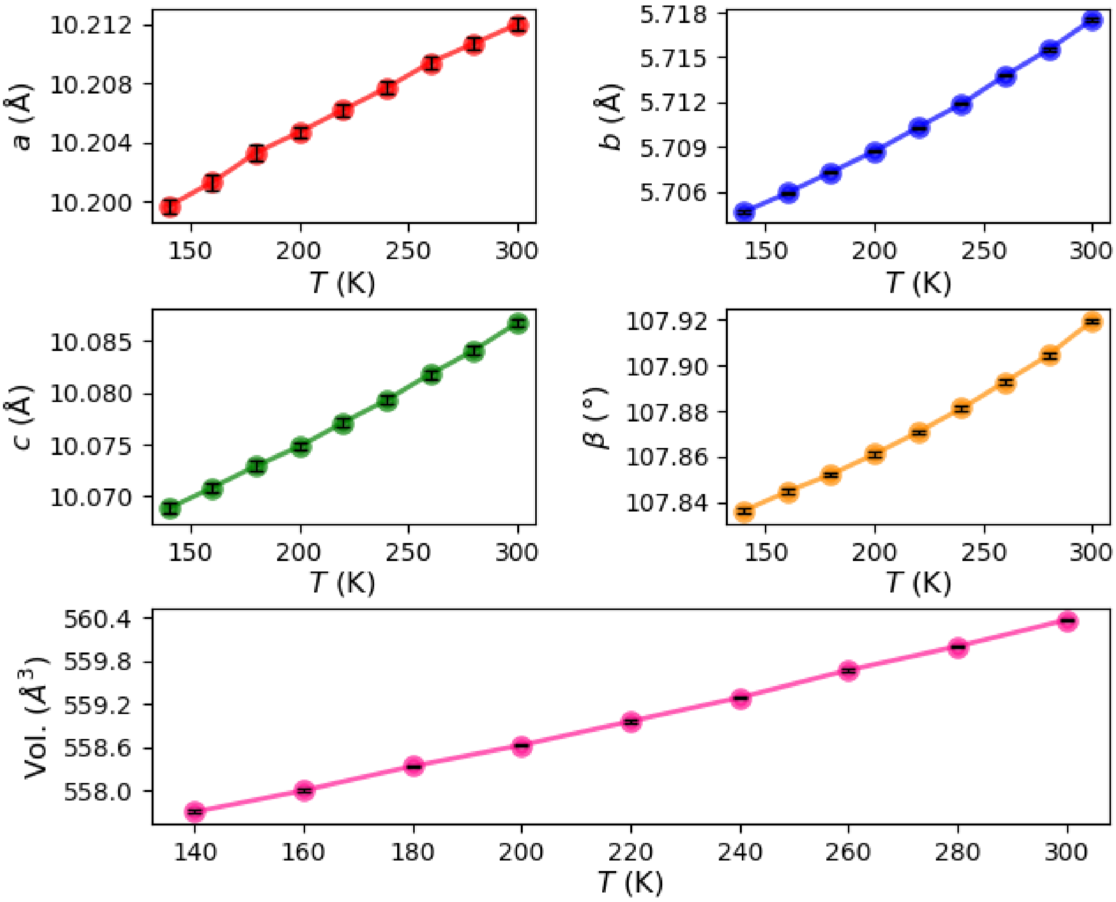

Figure S6. Unit cell parameters and unit cell volume of $Ba_2CuTe_{0.7}W_{0.3}O_6$ in the monoclinic phase, $C2/m$, as a function of *T* between 140-300 K.

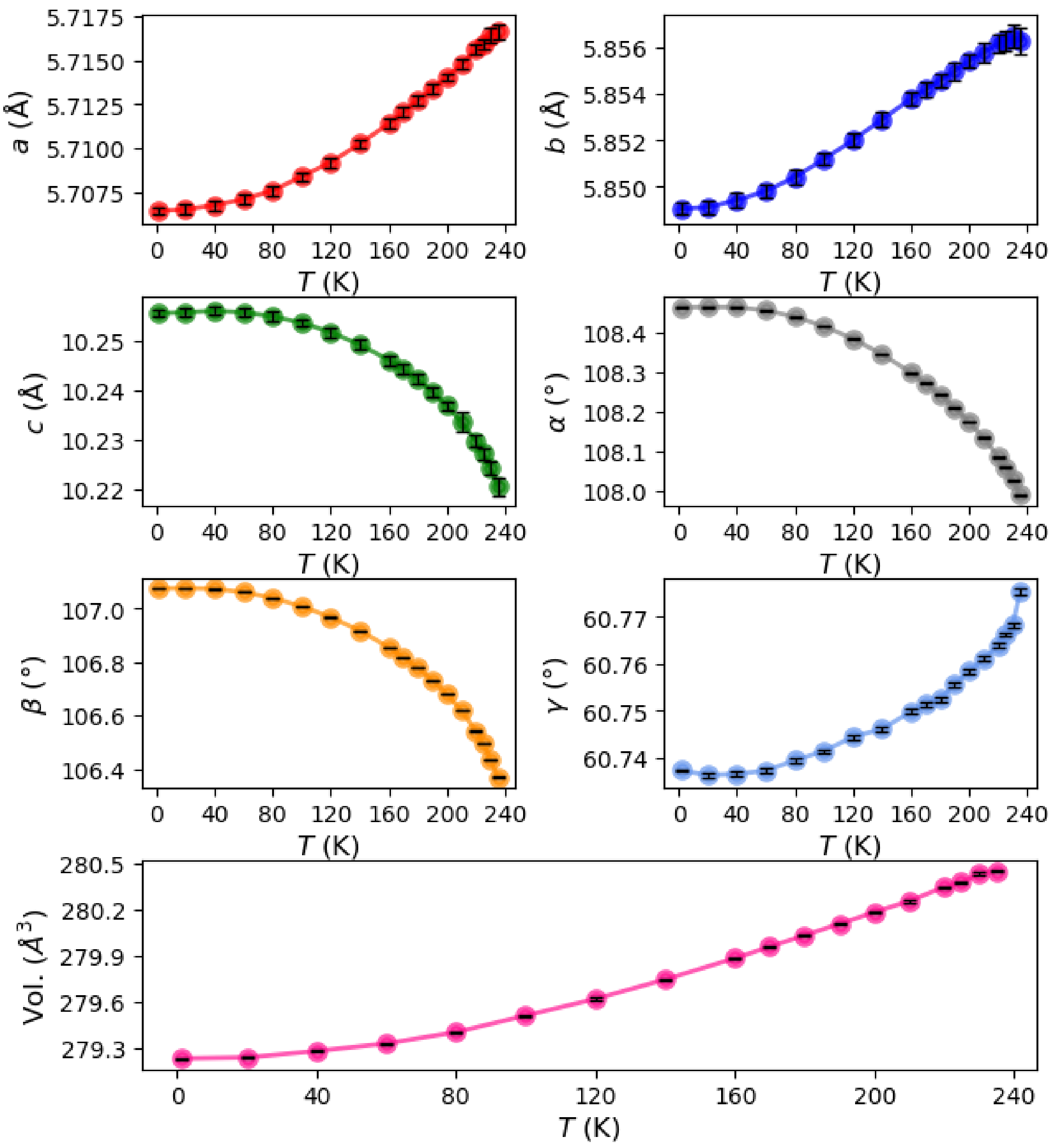

Figure S7. Unit cell parameters and unit cell volume of $Ba_2CuTe_{0.9}W_{0.1}O_6$ in the triclinic phase, $P\bar{1}$, as a function of *T* between 1.55-235 K.

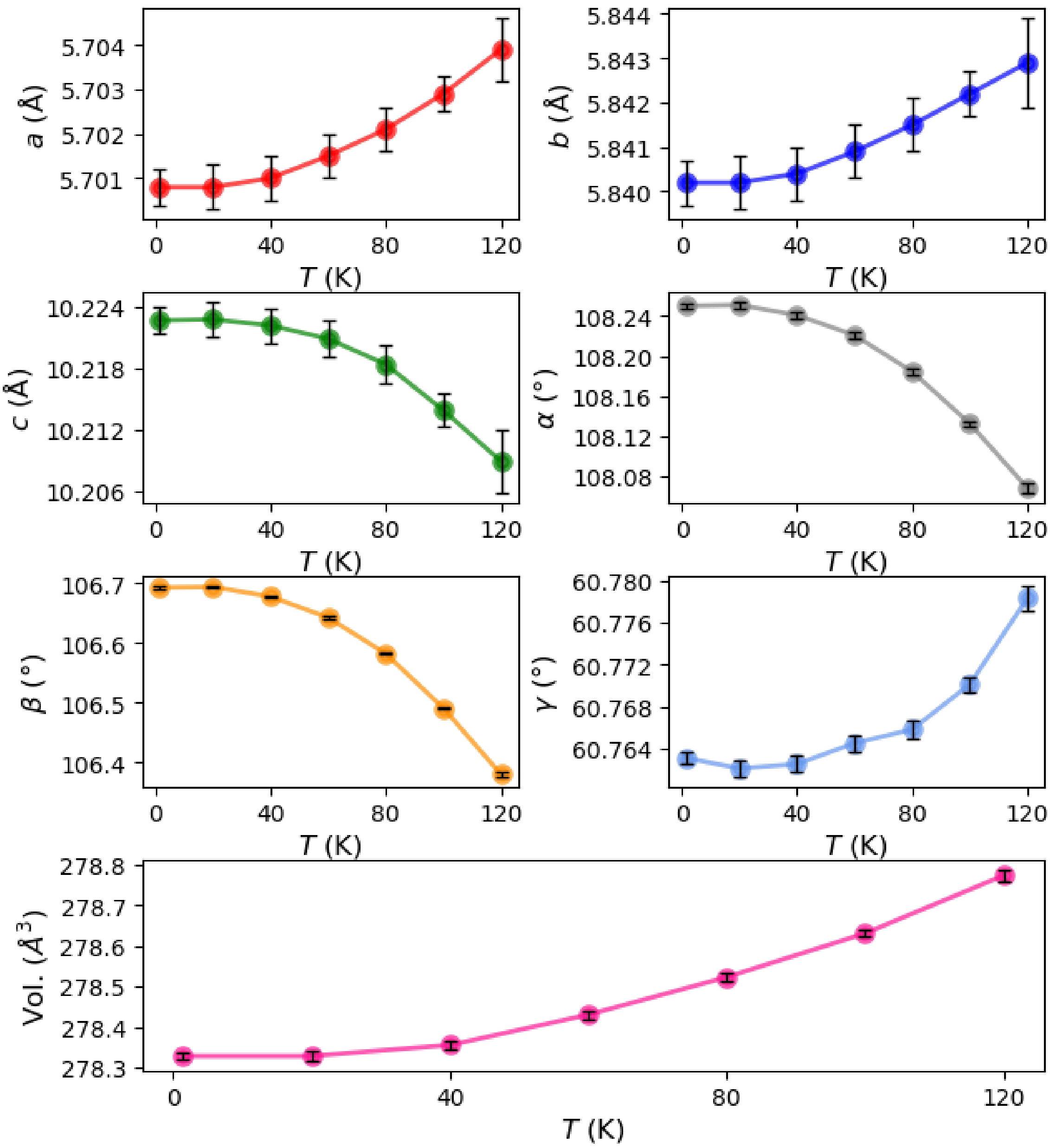

Figure S8. Unit cell parameters and unit cell volume of $Ba_2CuTe_{0.7}W_{0.3}O_6$ in the triclinic phase, $P\bar{1}$, as a function of T between 1.44-120 K.

b) Non-linear unit cell volume in triclinic phase

The unit cell volume changes non-linearly in the triclinic phase of both $Ba_2CuTe_{0.9}W_{0.1}O_6$ and $Ba_2CuTe_{0.7}W_{0.3}O_6$. Several possible reasons for this behaviour were investigated. Firstly, Jahn-Teller distortion of the CuO_6 octahedra within the triclinic phase was investigated with temperature. Figure S9 and S10 show little change in the distortion with temperature, with the Jahn-Teller distortion parameter (σ_{JT}) remaining approximately constant with T in both x = 0.1 and 0.3.

Jahn-Teller distortion parameter: $\sigma_{JT} = \sqrt{\frac{1}{6}\sum_i[(\mathrm{Cu}-\mathrm{O})_i - \langle \mathrm{Cu}-\mathrm{O}\rangle]^2}$ (1)

The Te(1)-Cu(1) and Te(2)-Cu(1) distances were then investigated. Figure S11 and S12 show the distances for $x = 0.1$ and $x = 0.3$, respectively. It was observed in both $x = 0.1$ and 0.3, the Te(1)-Cu(1) distances decreased with *T* which would contribute to a reduction in *a* and *b*, however not significantly enough to account for the total unit cell contraction in the *ab* plane. Similarly, it was observed there is very little change in the Te(2)-Cu(1) distance with temperature. In fact, the slight decrease in the Te(2)-Cu(1) distances within the Cu(1)-Te(2)-Cu(1) trimer should contribute towards a reduction in *c*.

The revelation comes from looking at the bond angles for the three Te/W(1)-O-Cu(1) corner-sharing linkers and the Cu(1)-O-Te(2) bond angle in the face-sharing Cu-Te-Cu trimer. The bond angles for $x = 0.1$ and 0.3 are shown in Figure S13 and S14. The Te/W(1)-O-Cu(1) bond angles are already significantly perturbed away from 180° in both the monoclinic and triclinic unit cells. However, as *T* is lowered, the distortion gets worse. The angle data for $Ba_2CuTe_{0.9}W_{0.1}O_6$ in Figure S13 shows the Cu(1)-O(5)-Te(1) bond angle decreases from 171.5° to 168.0° between 200-1.5 K - a change of Δ(°) ~ 3.4. The Cu(1)-O(4)-Te(1) bond angle is reduced by a similar angle of Δ(°) ~ 3.0 within this temperature range, while the Cu(1)-O(6)-Te(1) angle is reduced less by Δ(°)~ 0.8.

Alternatively, the Cu(1)-O(1-3)-Te(2) bond angles within the Cu-Te-Cu trimer change little with temperature. Reducing the Cu(1)-O-Te(2) bond angles would bring the positively charged face-sharing Te^{6+} and Cu^{2+} cations closer together. This would create a significant repulsion between the cation sites. Hence, the structure prefers to keep the face-sharing cations apart and minimize the reduction of the Te(2)-Cu(1) trimer distance. To do this, the face-sharing trimer acts like a rigid pole along the *c* axis that resists compression as *T* lowers. Instead, the reduction in the unit cell lengths is directed perpendicular to the trimer along *a* and *b* by reducing the Te/W(1)-O-Cu(1) bond lengths and decreasing the Te/W(1)-O-Cu(1) bond angles. The combined effect of both pushes the *ab* planes at the top and bottom of the triclinic unit cell up and down, respectively. Whilst this occurs, *a* and *b* decrease, and the unit cell is elongated along *c*. The Te/W(1)-O-Cu(1) bond angles do not alter by the same amount, with the Te/W(1)-O(4, 5)-Cu(1) angles decreasing more than the Te/W(1)-O(6)-Cu(1) angle. This has the effect of reducing γ but increasing β and α as the temperature is lowered. Therefore, the mixture of face- and corner-sharing leads to anisotropic thermal expansion of the unit cell. The competition between contraction along *a* and *b* and expansion along *c,* leads to a non-linear decrease in the unit cell volume of $x = 0.1$ and $x = 0.3$ with *T*.

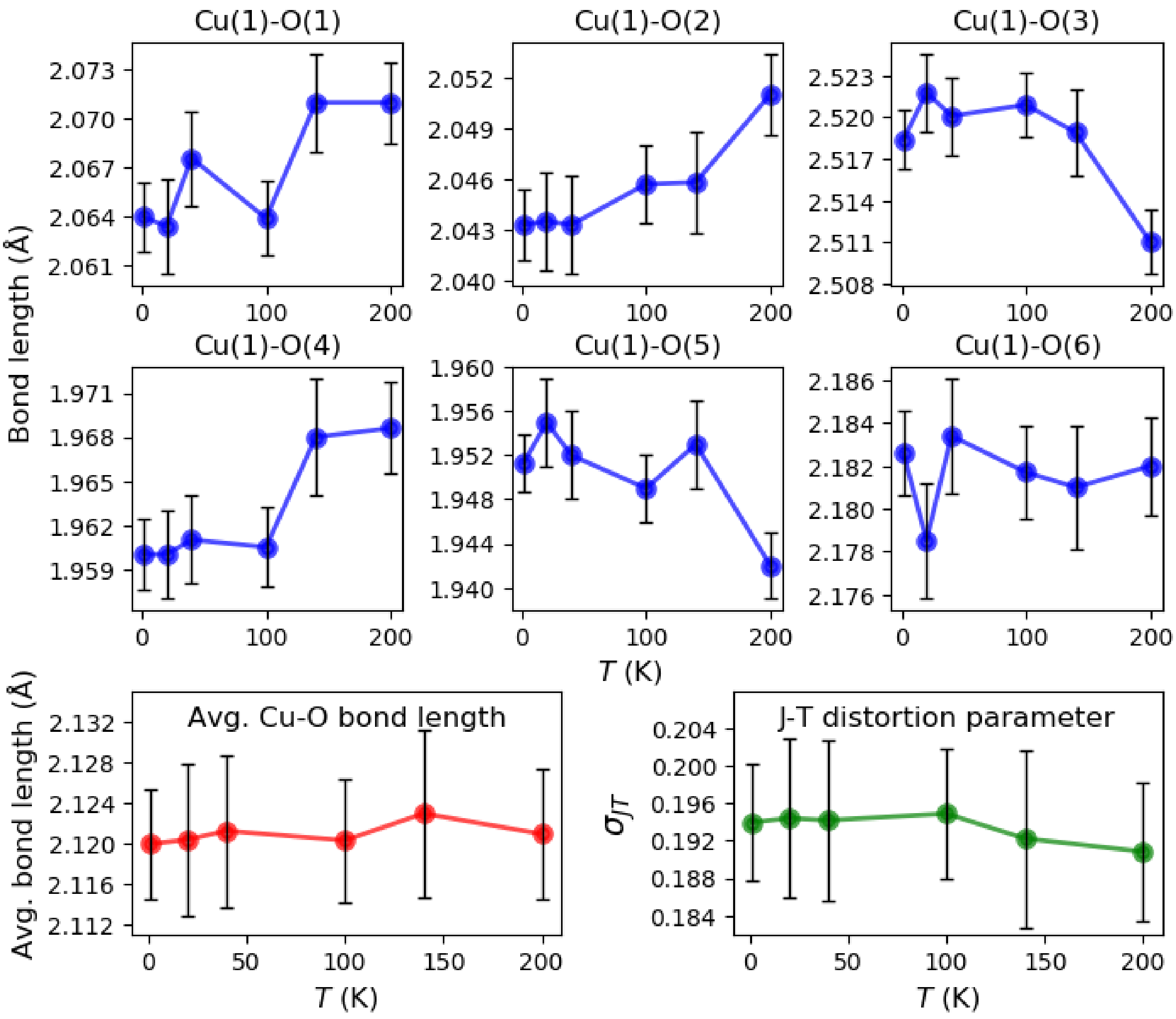

Figure S9. Cu(1)-O(1-6) bond lengths in $Ba2CuTe_{0.9}W_{0.1}O_6$ as a function of T in the $P\bar{1}$ phase. Also shown are the average Cu-O bond length and the Jahn-Teller distortion parameter.

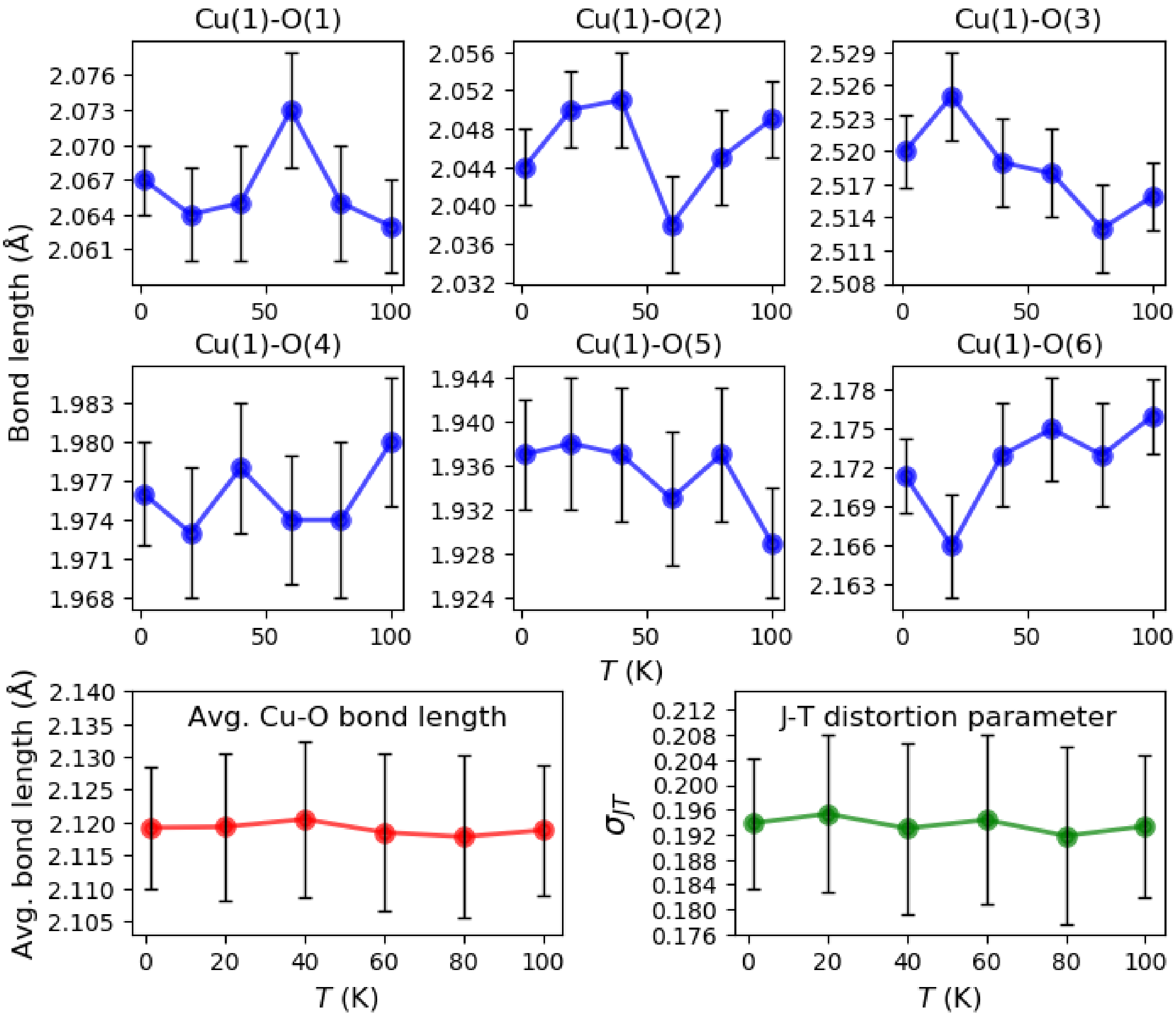

Figure S10. Cu(1)-O(1-6) bond lengths in $Ba2CuTe_{0.7}W_{0.3}O_6$ as a function of T in the $P\bar{1}$ phase. Also shown are the average Cu-O bond length and the Jahn-Teller distortion parameter.

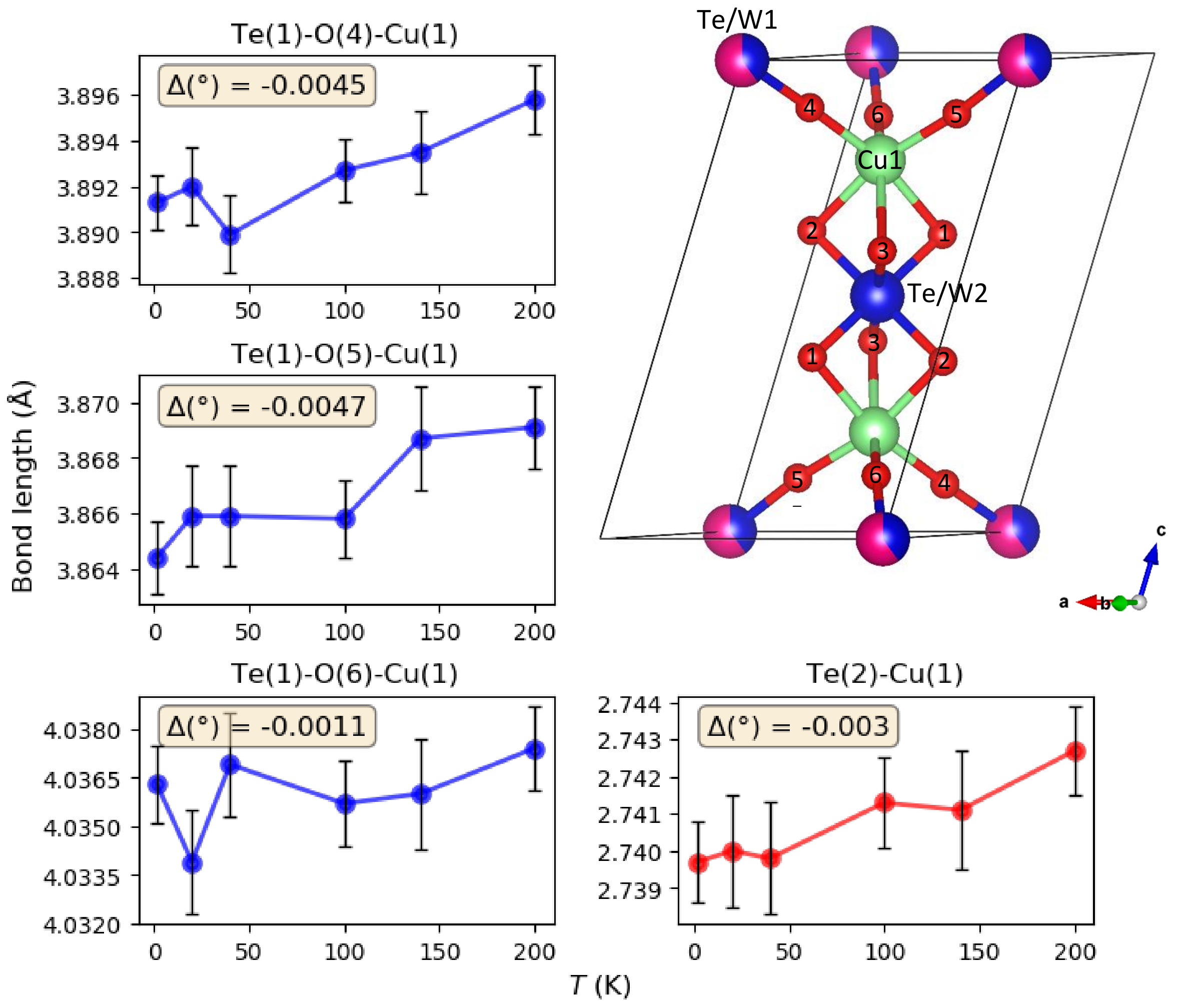

Figure S11. Te(1)-Cu(1) and Te(2)-Cu(1) distances in $Ba_2CuTe_{0.9}W_{0.1}O_6$ in the $P\bar{1}$ phase.

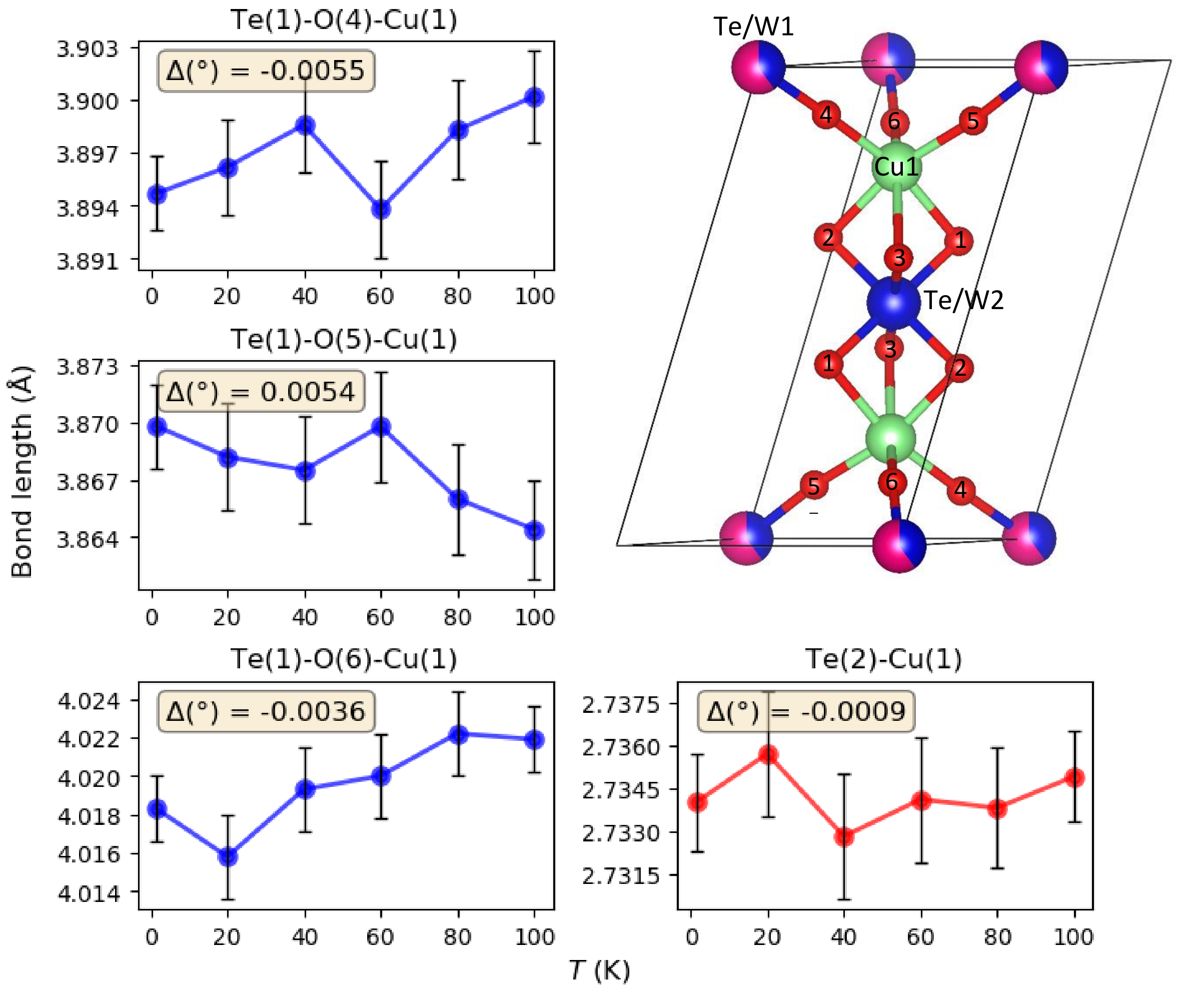

Figure S12. Te(1)-Cu(1) and Te(2)- Cu(1) distances in $Ba_2CuTe_{0.7}W_{0.3}O_6$ in the $P\bar{1}$ phase.

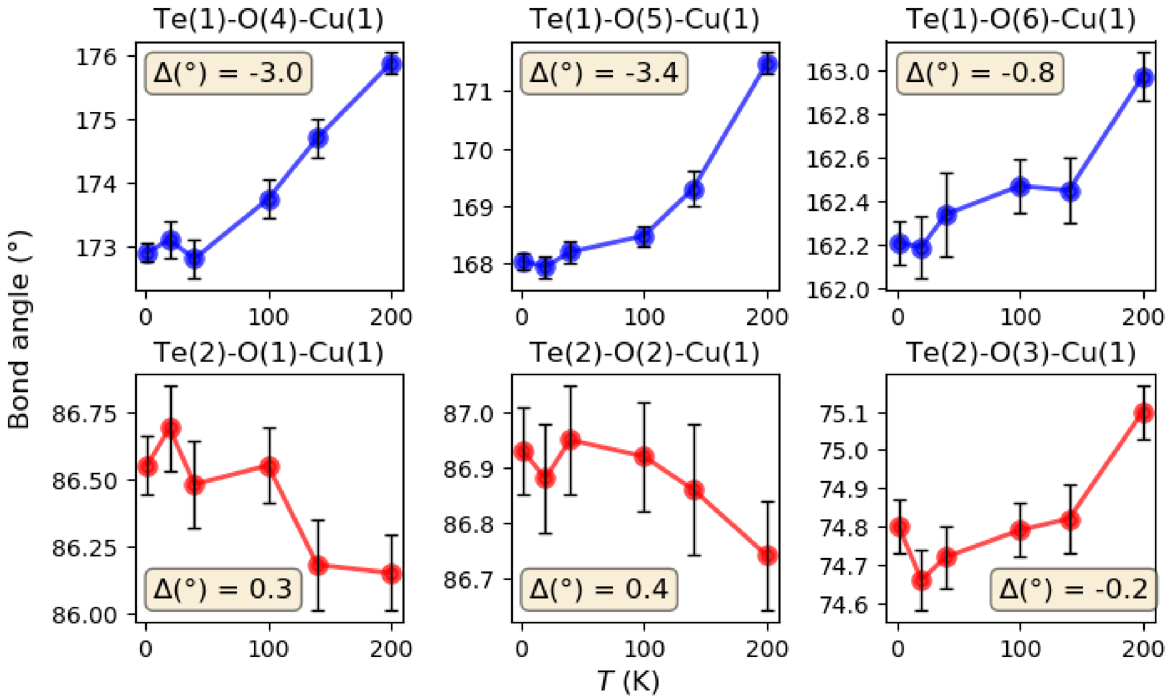

Figure S13. Te(1, 2)-O(6-1)-Cu(1) bond angles in $Ba_2CuTe_{0.9}W_{0.1}O_6$ in the $P\bar{1}$ phase.

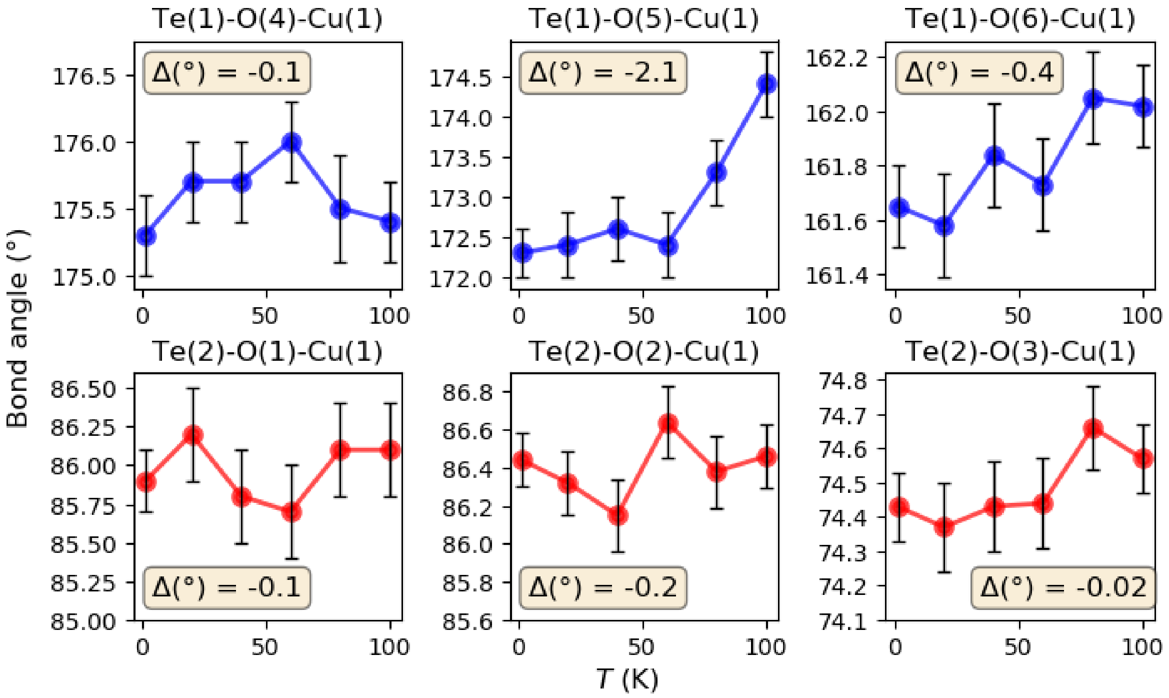

Figure S14. Te(1, 2)-O(6-1)-Cu(1) bond angles in $Ba_2CuTe_{0.7}W_{0.3}O_6$ in the $P\bar{1}$ phase.

c) Jahn-Teller distortion of the CuO_6 octahedra in $Ba_2CuTe_{09}W_{0.1}O_6$ and $Ba_2CuTe_{0.7}W_{0.3}O_6$

Jahn-Teller distortion was measured empirically using the J-T distortion parameter (σ_{JT}) below:

$$\sigma_{JT} = \sqrt{\frac{1}{6}\sum_i[(\mathrm{Cu}-\mathrm{O})_i - \langle \mathrm{Cu}-\mathrm{O}\rangle]^2} \qquad (2)$$

$(\mathrm{Cu}-\mathrm{O})_i$ represents the Cu-O neutron bond length on the i-Cu(1)O_6 site and $\langle \mathrm{Cu}-\mathrm{O}\rangle$ is the mean bond length. σ_{JT} is plotted as a function of *T* for *x* = 0.1 and *x* = 0.3 in Figure S15, where the T_{trans} ranges are marked on the graph. The equatorial bond lengths are approximately equal for any given temperature. The distortion parameter increases slightly with decreasing *T* for both *x* = 0.1 and *x* = 0.3, reflecting further gradual distortion of CuO_6 on cooling from 300 to 100 K, with no large discontinuities. There is no further distortion on cooling below 100 K. Within the monoclinic phase, *x* = 0.3 has a consistently lower value of σ_{JT}. The Cu-O bond distances in Figure S16 support this with less elongation of the axial Cu-O bonds for the *x* = 0.3 composition and a smaller variance in the Cu-O bond lengths.

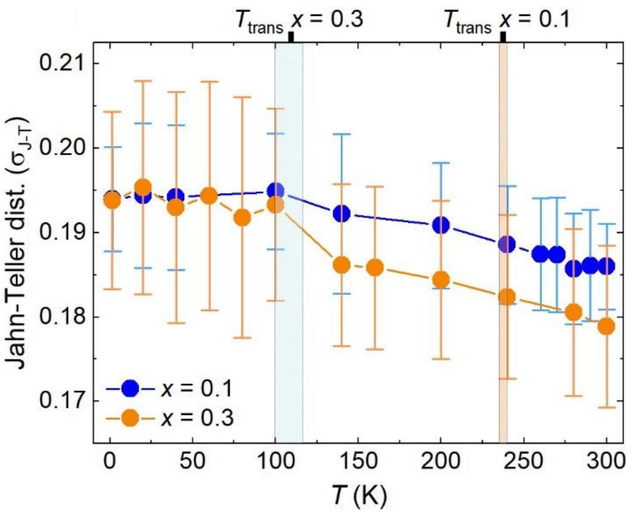

Figure S15. The Jahn-Teller distortion parameter (σ_{JT}) as a function of temperature (*T*) for *x* = 0.1 and *x* = 0.3. The T_{trans} ranges for the monoclinic to triclinic transition on cooling are marked in the plot.

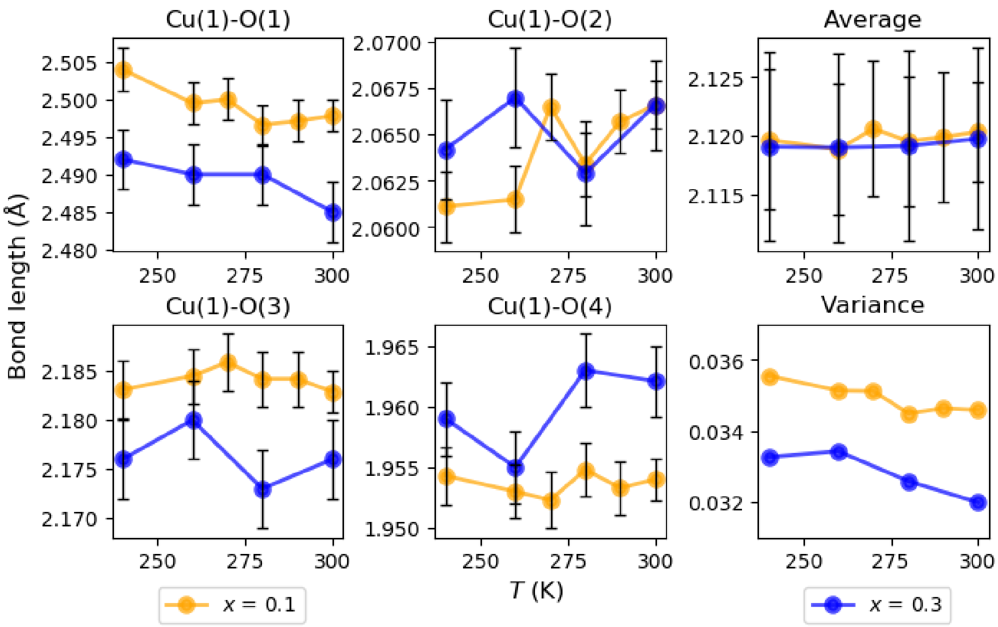

Figure S16. Cu(1)-O(1-4) bond lengths in $Ba_2CuTe_{0.9}W_{0.1}O_6$ and $Ba_2CuTe_{0.9}W_{0.1}O_6$ in the $C/2m$ phase.

3. Synchrotron X-ray diffraction

Synchrotron X-ray diffraction patterns were collected using a wavelength of λ = 0.20742 Å at 300 K for the following three samples: $Ba_2CuTe_{0.7}W_{0.3}O_6$, $Ba_2CuTe_{0.8}W_{0.2}O_6$ and $Ba_2CuTe_{0.9}W_{0.1}O_6$. To determine the W^{6+} site occupancy, the refinements were performed using three possible monoclinic models. These models were: (1) W^{6+} exclusively on the *B*''(c) site; (2) W^{6+} exclusively on the *B*''(f) site; and (3) W^{6+} shared equally between the *B*''(c) and *B*''(f) sites. Tables S5, S6 and S7 below show the *R*-values obtained for each sample using these models. For every sample, the model with the lowest *R*-values is model (1) where W^{6+} exclusively occupies the *B*''(c) corner sharing site. Occupancy of the *B*''(f) site by W^{6+} produces a poorer fit in comparison and leads to negative Uiso values indicating an unstable refinement.

The *B*''(c) and *B*''(f) site fractions in model (1) were refined using the appropriate constraints for the structural composition. The highly correlated Uisos for the *B*''(c) and *B*''(f) sites were turned off during the refinement. During the refinement, a small fraction of W^{6+} moved onto the *B*''(f) site. For each composition, the percentage of the total amount of W^{6+} in $Ba_2CuTe_{1-x}W_xO_6$ residing on the *B*''(f) site was calculated by dividing the *B*''(f)-W2 site fraction by the *B*''(c)-W1 site fraction. The percentage of W^{6+} residing on the *B*''(f) site was found to be: 4.7(2)% ($Ba_2CuTe_{0.8}W_{0.2}O_6$); 4.7(2)% ($Ba_2CuTe_{0.8}W_{0.2}O_6$); and 5.3(2)% ($Ba_2CuTe_{0.7}W_{0.3}O_6$). Tables S8, S9 and S10 show the refined crystal structures. Placing ~ 5% of W^{6+} in each composition on the *B*''(f) site provides a slightly improved description of the crystal structures. To check the stability of the refinement, the refined site fractions were changed to those of model (2) i.e. exclusive occupancy of the *B*''(f) site. Repeating the refinement converged to the same result as when approaching the refinement using site fractions from model (1) in the initial structural model. This showed a strong preference for W^{6+} doping on the *B*''(c) site.

Table S5: R-values obtained from refinement of synchrotron X-ray data for $Ba_2CuTe_{0.7}W_{0.3}O_6$ using different W^{6+} site occupancy models

Site occupancy	R_{wp} (%)	R_p (%)	R_{exp} (%)	χ^2
W^{6+} exclusively on *B*''(c) site	2.65	1.88	0.81	10.69
W^{6+} exclusively on *B*''(f) site	4.81	3.35	0.81	35.28
W^{6+} equal (50:50) on both *B*''(c) and *B*''(f) sites	3.14	2.25	0.81	14.98

Table S6: R-values obtained from refinement of synchrotron X-ray data for $Ba_2CuTe_{0.8}W_{0.2}O_6$ using different W^{6+} site occupancy models

Site occupancy	R_{wp} (%)	R_p (%)	R_{exp} (%)	χ^2
W^{6+} exclusively on *B*''(c) site	1.76	1.34	0.82	4.58
W^{6+} exclusively on *B*''(f) site	3.10	2.23	0.82	14.29
W^{6+} equal (50:50) on both *B*''(c) and *B*''(f) sites	2.07	1.57	0.82	6.35

Table S7: R-values obtained from refinement of synchrotron X-ray data for $Ba_2CuTe_{0.9}W_{0.1}O_6$ using different W^{6+} site occupancy models

Site occupancy	R_{wp} (%)	R_p (%)	R_{exp} (%)	χ^2
W^{6+} exclusively on *B*''(c) site	1.54	1.13	0.84	3.42
W^{6+} exclusively on *B*''(f) site	2.03	1.47	0.84	5.86
W^{6+} equal (50:50) on both *B*''(c) and *B*''(f) sites	1.65	1.21	0.84	3.88

Table S8: $Ba_2CuTe_{0.7}W_{0.3}O_6$ (x = 0.3) structure when site occupancy refined

Space Group: $C2/m$, No. 12, 300 K<br>R_P = 1.84%, R_{wp} = 2.60%, R_{exp} = 0.81%, χ^2 = 10.50, var. 86<br>a = 10.2090(3) Å, b = 5.71596(6) Å, c = 10.08392(33) Å, β = 107.9255(9)°<br>*Vol.* = 559.876(13) Å^3						
Site	Wyckoff Position	x	y	z	Site fraction	Uiso (Å^2)
Ba1	4i	0.12949(10)	0	0.37951(7)	1.0	0.00715(23)
Ba2	4i	0.28386(11)	0	0.85026(7)	1.0	0.01104(25)
Te1	2a	0	0	0	0.430(1)	0.00808
W1	2a	0	0	0	0.570(1)	0.00808
Te2	2d	0	0.5	0.5	0.970(1)	0.00358
W2	2d	0	0.5	0.5	0.030(1)	0.00358
Cu1	4i	-0.09429(20)	0.5	0.21528(15)	1.0	0.01029(47)
O1	4i	0.13200(11)	0.5	0.40293(83)	1.0	0.03453(276)
O2	8j	-0.10944(57)	0.73097(83)	0.36866(43)	1.0	0.00071(132)
O3	4i	0.31263(84)	0.5	0.87764(76)	1.0	0.00932(213)
O4	8j	0.04436(76)	0.75009(13)	0.88975(56)	1.0	0.01433(131)

Table S9: $Ba_2CuTe_{0.8}W_{0.2}O_6$ (x = 0.2) structure when site occupancy refined

Space Group: $C2/m$, No. 12, 300 K<br>R_P = 1.34%, R_{wp} = 1.75%, R_{exp} = 0.82%, χ^2 = 4.54, var. 86<br>a = 10.21742(26) Å, b = 5.7173(4) Å, c = 10.08907(25) Å, β = 107.9346(7)°<br>*Vol.* = 560.768(10) Å^3						
Site	Wyckoff Position	x	y	z	Site fraction	Uiso (Å^2)
Ba1	4i	0.12946(7)	0	0.37943(5)	1.0	0.00750(17)
Ba2	4i	0.28370(8)	0	0.85019(5)	1.0	0.01068(18)
Te1	2a	0	0	0	0.618(1)	0.00666
W1	2a	0	0	0	0.382(1)	0.00666
Te2	2d	0	0.5	0.5	0.982(1)	0.00396
W2	2d	0	0.5	0.5	0.018(1)	0.00369
Cu1	4i	-0.09399(14)	0.5	0.2151(1)	1.0	0.0094(3)
O1	4i	0.1330(8)	0.5	0.4024(6)	1.0	0.02412(178)
O2	8j	-0.1077(4)	0.7310(6)	0.3687(3)	1.0	0.00361(97)
O3	4i	0.3145(6)	0.5	0.8746(5)	1.0	0.00652(149)
O4	8j	0.04697(5)	0.7538(9)	0.8905(4)	1.0	0.01459(93)

Table S10: $Ba_2CuTe_{0.9}W_{0.1}O_6$ (x = 0.1) structure when site occupancy refined

Space Group: $C2/m$, No. 12, 300 K<br>R_P = 1.13%, R_{wp} = 1.54%, R_{exp} = 0.4%, χ^2 = 3.39, var. 86<br>a = 10.2247(2) Å, b = 5.72007(4) Å, c = 10.09322(23) Å, β = 107.9570(9)°<br>*Vol.* = 559.876(13) Å^3						
Site	Wyckoff Position	x	y	z	Site fraction	Uiso (Å^2)
Ba1	4i	0.12936(6)	0	0.37930(4)	1.0	0.00786(15)
Ba2	4i	0.28331(7)	0	0.85007(4)	1.0	0.00984(16)
Te1	2a	0	0	0	0.809(1)	0.00567
W1	2a	0	0	0	0.191(1)	0.00567

Te2	2d	0	0.5	0.5	0.991(1)	0.00497
W2	2d	0	0.5	0.5	0.009(1)	0.00497
Cu1	4i	-0.09367(12)	0.5	0.21510(9)	1.0	0.00827(28)
O1	4i	0.13281(62)	0.5	0.40094(45)	1.0	0.01419(141)
O2	8j	-0.10638(37)	0.73184(56)	0.36854(28)	1.0	0.00675(89)
O3	4i	0.31591(51)	0.5	0.87345(44)	1.0	0.00737(133)
O4	8j	0.04899(49)	0.75696(80)	0.89113(35)	1.0	0.01453(83)

4. Extended X-ray Absorption Fine Structure (EXAFS) data for $Ba_2CuTe_{0.7}W_{0.3}O_6$

The site occupancy of W^{6+} within the monclinic structre of $Ba_2CuTe_{0.7}W_{0.3}O_6$ was further investigated by analysis of W L_3 edge EXAFS data. Models of the local environment assumed: (1) full W^{6+} substitution on the *B*''(c) site; and (2) full W^{6+} substitution on the *B*''(f) site. As shown below, data were adequately fitted without inclusions of nearest neighbour W…Te paths, and, therefore, potential W…W scattering paths could be reasonably neglected.

Model (1) provides a plausible environment for W^{6+} doping on the *B*''(c) site. As summarised in Table S11, all significant scattering paths within a radial distance of 4 Å from the W absorber are fitted, with reasonable contact distances and positive Debye-Waller factors. The EXAFS determined W-O bond length (1.900(7) Å) is close to the average Te/W-O bond length on the Te/W(1)O_6 site (1.920(5) Å) determined from the 300 K neutron diffraction data. It should be noted there is a slight variance in the Te/W(1)-O bond lengths (owing to the low symmetry) with the W/Te(1)-O(3) length being 1.902(2) Å and the W/Te(1)-O(4) being slightly longer at 1.930(2) Å. This difference is too small to be observed using EXAFS. The Bond Valence Sum determined for the W environment was 6.3(8) v.u.[2], consistent with the expected oxidation state. Figure S17 compares shows the calculated model fit in comparison with the experimental data as $k^2\chi(k)$ and $\chi(R)$, its Fourier transform. The model clearly affords an excellent fit to the experimental data, as evident by the graphical fit and low R-factor of 1.18% (Table S11).

In contrast model (2), fails to provide a plausible description for W^{6+} doping on the *B*''(f) site. As shown in Table S12, although model (2) affords a reasonable W(Te2)…O3 path length, the associated uncertainty is two orders of magnitude greater than determined for Model 1, and the Debyer-Waller factor is negative. Several other refined path lengths also have negative Debye-Waller factors, as shown in Table S12. The graphical comparison of the calculated model fit and experimental data, Figure 19, shows noticeably poor agreement in several regions, which is reflected in the high R-factor of 9.07%. Consideration of the contribution of individual paths to the $\chi(R)$ data, show that the region $3<R<4$ Å region is only well fitted by the W(Te1)…O4.1, W(Te1)…O4.1…Cu1.1, and W(Te1)…O4.1…Cu1.1…O4.1 paths afforded by W^{6+} doping on the Te(1) site as shown in Figure S18. In contrast, the local environment of W^{6+} doping on the *B*''(f) site does not afford scattering paths to adequately fit the data in this range, as shown in Figure S20.

The possibility of W^{6+} substitution of the Cu was also examined, however the model failed to provide a physically credible solution. In particular, the S_0^2 parameter, which accounts for relaxation of the absorber atom in the presence of the core hole refined to a negative, and hence meaningless, value; for transmission measurements a value in the range $0.7 < S_0^2 < 1.0$ is normally expected. In addition, refinement of some path lengths converged to implausible values or had associated Debye-Waller factors which were negative.

Our analysis of W L_3 EXAFS data therefore provides evidence for preferential W^{6+} substitution on the *B*''(c) site, in agreement with the diffraction data, but has the advantage of providing an element specific perspective. Attempts were made to fit the data using contributions from both models (1) and (2), under linear restraints, to assess the potential for disorder of a fraction of W^{6+} from the *B*''(c) to *B*''(f) site. However, it was not possible to adequately stabilise such a fit, since the number of variables approached the number of data points. Yet, there is some evidence for a contribution from the model (2) when comparing the $\chi(R)$ EXAFS plot in the range 2.0 < R < 3.0. In Figure S17, there is a slight intensity mismatch between the fit and experimental data for the peaks in at a radial distance of ~2.1 and ~2.7 Å. In contrast, these same peaks are described quite well in Figure S19 where the model (2) structure is used. Therefore, it is feasible this intensity mismatch in Figure S17 between 2.0 < R < 3.0 represents the contribution from the *B*''(f) site which could not be stabilised in the fit given low 5% W^{6+} site occupancy shown in the synchrotron X-ray data.

Table S11: Refined model parameters for W substitution on Te(1) site of $Ba_2CuTe_{0.7}W_{0.3}O_6$. Note that the path descriptions are those generated by the ATOMS algorithm to define the local cluster within Artemis.[3] R is the refined path length of the specified path and σ^2 is the EXAFS Debye-Waller factor; N is the path degeneracy, which corresponds to the co-ordination number in the first shell. The global parameters are: S_0^2, the passive electron reduction factor; the energy alignment factor, ΔE_0; the number of independent data points, according to the Nyquist criterion, N_{idp}; and the number of variables in the model, N_{var}. The fitted ranges were 3.0 < k < 11.0, with a Hanning window of dk = 1.0 Å^{-1}; and 1.15 < R < 4.0.

Shell	Path	N	R(Å)	σ^2(Å^2)	Global parameters
1	W(Te1)…O3.1 (single scattering)	6	1.900(7)	0.002(1)	S_0^2 = 0.76(6)<br>ΔE_0 = 7.3(1.0) eV<br>N_{idp} = 14<br>N_{var} = 8<br>$R(\%)$ =1.18
2	W(Te1)…O3.1…O4.1 (double scattering)	20	3.24(13)	0.003(2)	
2	W(Te1)…Ba2.1 (single scattering)	8	3.61(14)	0.007(3)	
2	W(Te1)…O3.1 (hinge)	24	3.80(15)	0.001(1)	
2	W(Te1)…O4.1 (forward though absorber)	4	3.82(15)	0.002(5)	
2	W(Te1)…O4.1…Cu1.1 (forward scattering)	8	3.85(15)	0.008(2)	
2	W(Te1)…O4.1…Cu1.1…O4.1 (double forward scattering)	4	3.85(15)	0.006(3)	

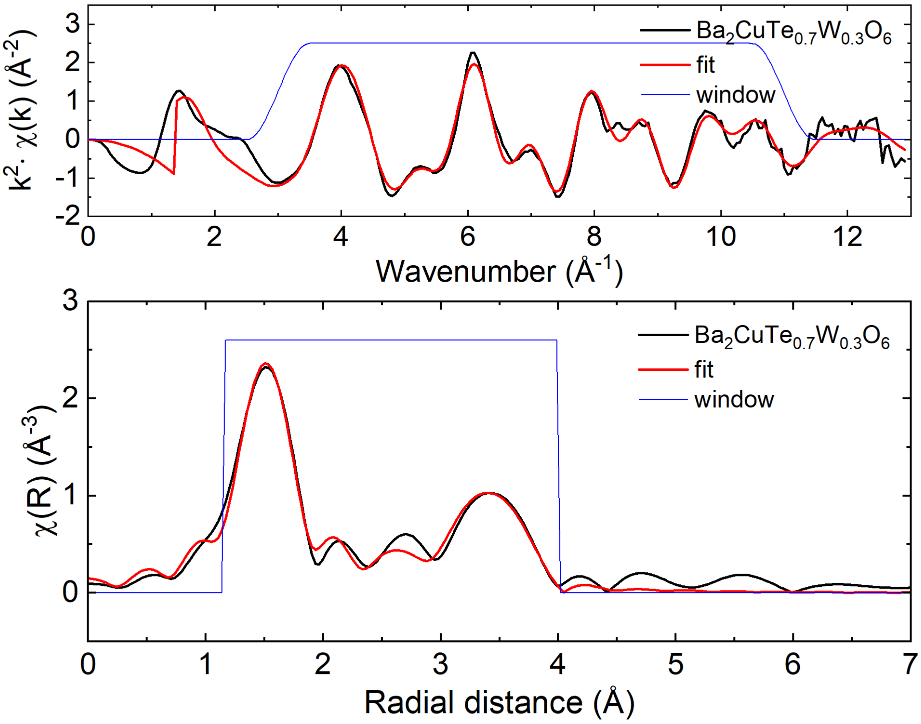

Figure S17. $k^2\chi(k)$ and $\chi(R)$ W L_3 EXAFS data of $Ba_2CuTe_{0.7}W_{0.3}O_6$ with model (1), assuming W^{6+} doping on Te(1) site (uncorrected for phase shift). Solid black lines represent experimental data and red lines represent the model fits. Fitting windows are indicated by solid blue lines.

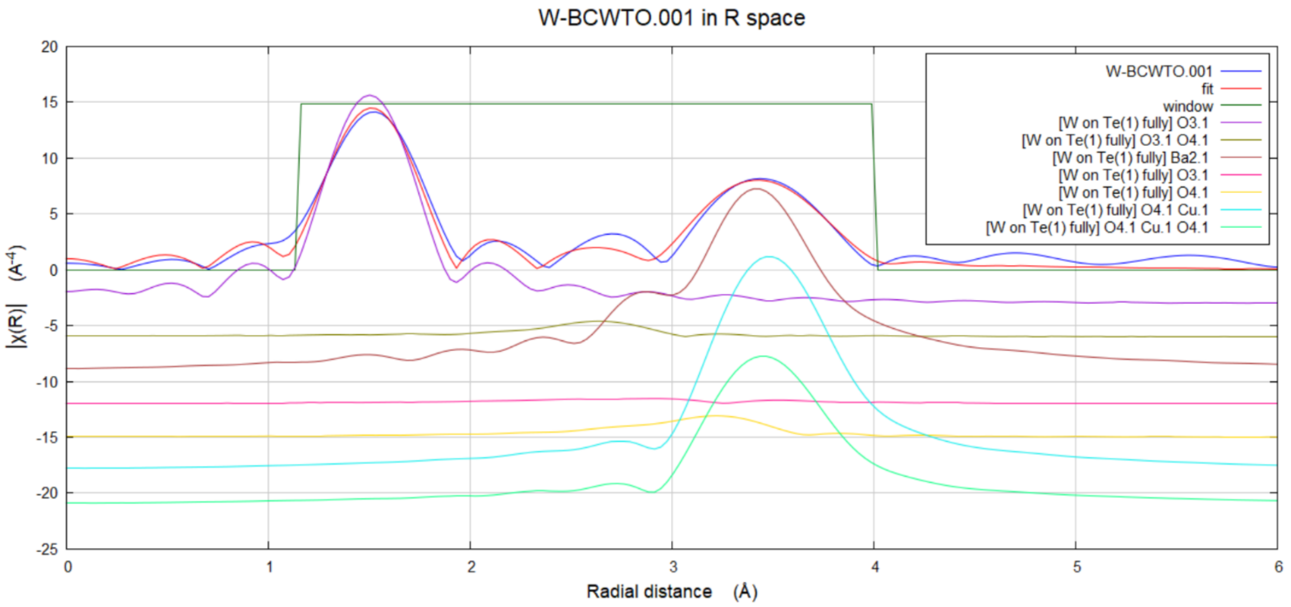

Figure S18. Showing component path contributions to $\chi(R)$ W L_3 EXAFS data of $Ba_2CuTe_{0.7}W_{0.3}O_6$ with model (1), assuming W substiution on Te(1) site (uncorrected for phase shift). Solid blue line represents experimental data and red line represents the model fit; other solid lines represent individual path contribution summarised in Table S11. The fitting window is shown indicated by the solid green line.

Table S12: Refined model parameters for W substitution on Te(2) site. Note that the path descriptions are those generated by the ATOMS algorithm to define the local cluster within Artemis.[3] R is the refined path length of the specified path and σ^2 is the EXAFS Debye-Waller factor; N is the path degeneracy, which corresponds to the co-ordination number in the first shell. The global parameters are: S_0^2, the passive electron reduction factor; the energy alignment factor, ΔE_0; the number of independent data points, according to the Nyquist criterion, N_{idp}; and the number of variables in the model, N_{var}. The fitted ranges were 3.0 < k < 11.0, with a Hanning window of dk = 1.0 Å^{-1}; and 1.15 < R < 4.5.

Shell	Path	N	R(Å)	σ^2(Å^2)	Global parameters
1	W(Te1)…O1.1 (single scattering)	6	1.90(15)	-0.0004(25)	S_0^2 = 0.62(13)<br>ΔE_0 = 7.6(2.3) eV<br>N_{idp} = 14<br>N_{var} = 8<br>R(%) =9.07
2	W(Te1)…Cu1.1 (double scattering)	2	2.69(21)	0.03(10)	
2	W(Te1)…O1.1…O2.1 (double scattering)	16	3.24(26)	-0.006(12)	
2	W(Te1)…Ba1.1 (single scattering)	4	3.45(27)	0.03(0.16)	
2	W(Te1)…O1.1 (forward though absorber)	6	3.80(30)	-0.012(3)	
2	W(Te1)…O2.1…Ba2.2 (non-forward linear)	8	4.69(37)	0.008(0.012)	

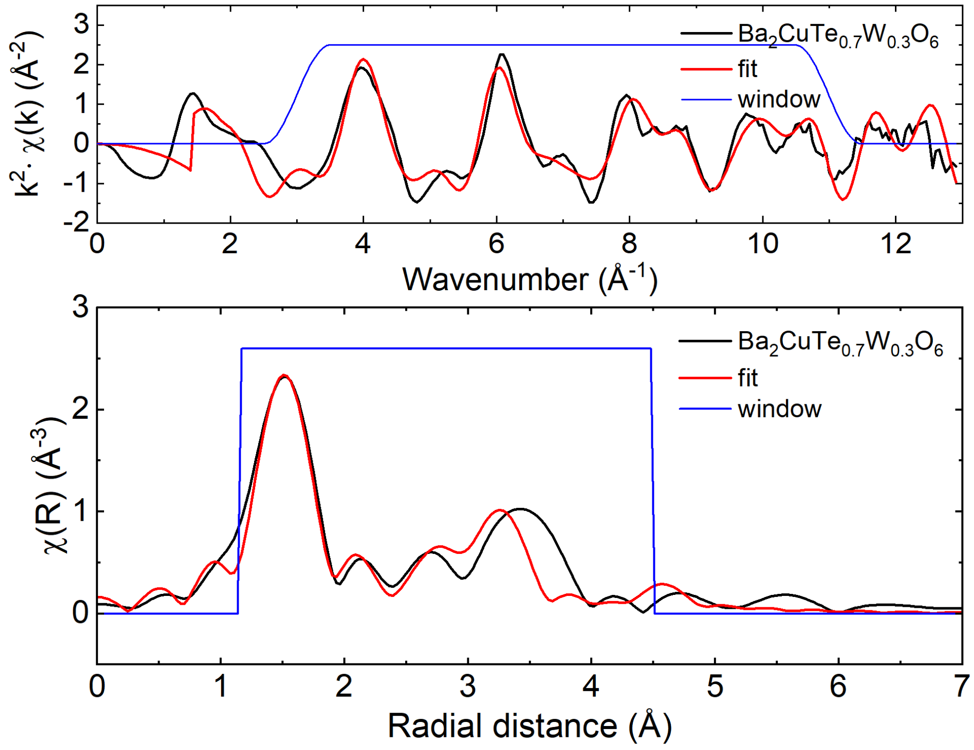

Figure S19. $k^2\chi(k)$ and $\chi(R)$ W L_3 EXAFS data of $Ba_2CuTe_{0.7}W_{0.3}O_6$ with model (2), assuming W^{6+} doping on Te(2) site (uncorrected for phase shift). Solid black lines represent experimental data and red lines represent the model fits. Fitting windows are indicated by solid blue lines.

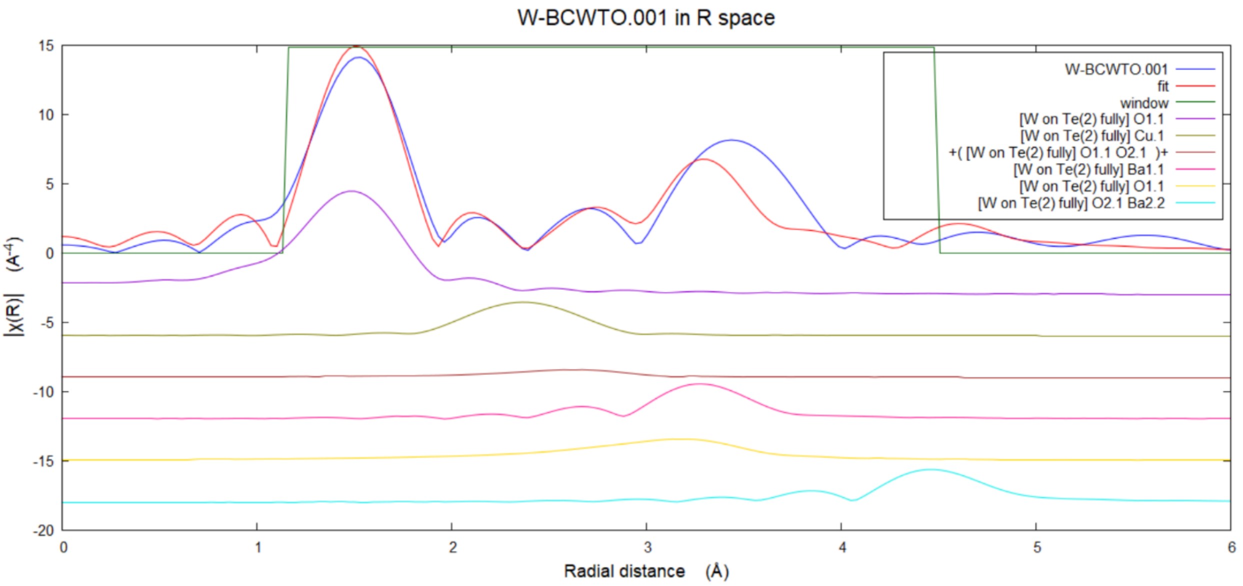

Figure S20. Showing component path contributions to $\chi(R)$ W L_3 EXAFS data of $Ba_2CuTe_{0.7}W_{0.3}O_6$ with model (2), assuming W^{6+} doping on Te(2) site (uncorrected for phase shift). Solid blue line represents experimental data and red line represents the model fit; other solid lines represent individual path contribution summarised in Table S12. The Fitting window is shown indicated by the solid green line.

5. **Magnetic Susceptibility**

(a) Curie-Weiss fitting

All the data were fitted to the inverse Curie-Weiss law $1/\chi= (T-\theta_W)/C$ between 200-400 K. Below are the Curie-Weiss fits for all the samples.

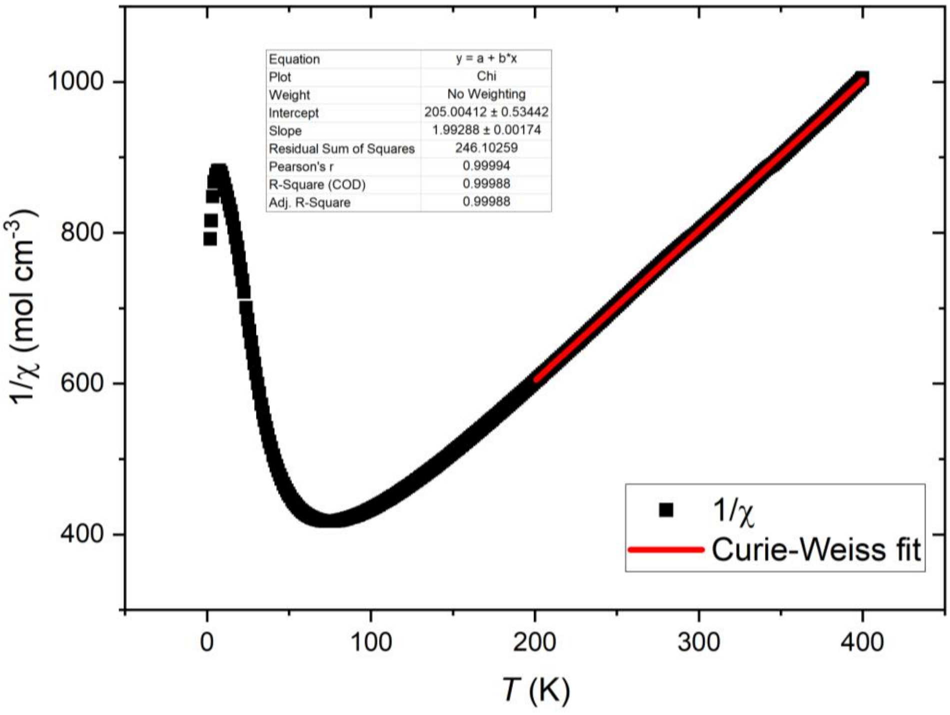

Figure S21. Curie-Weiss fit of Ba_2CuTeO_6 (x = 0)

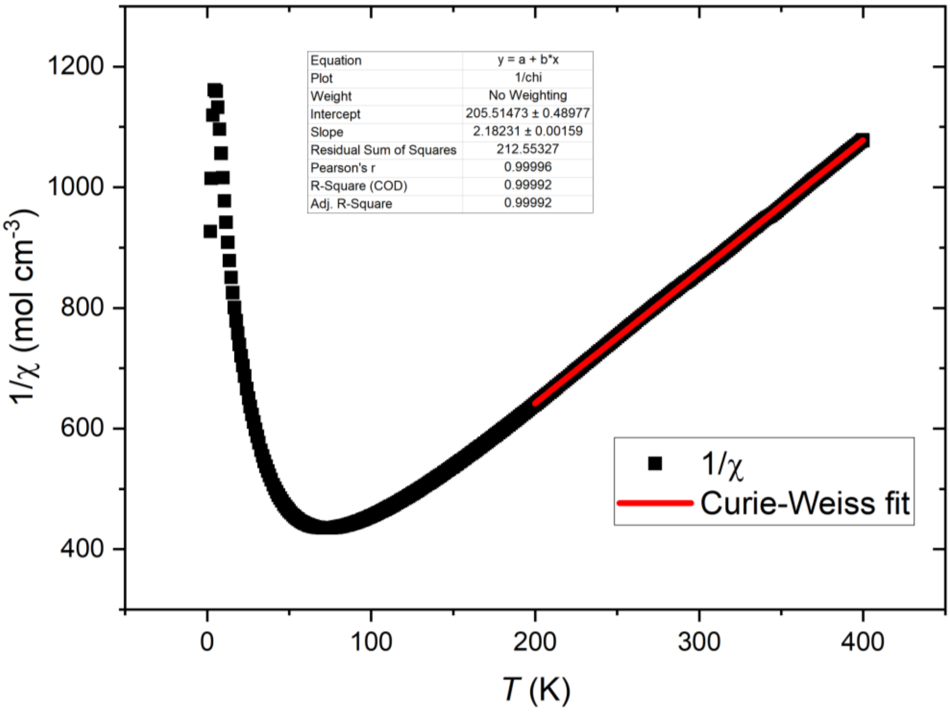

Figure S22. Curie-Weiss fit of $Ba_2CuTe_{0.95}W_{0.05}O_6$ (x = 0.05)

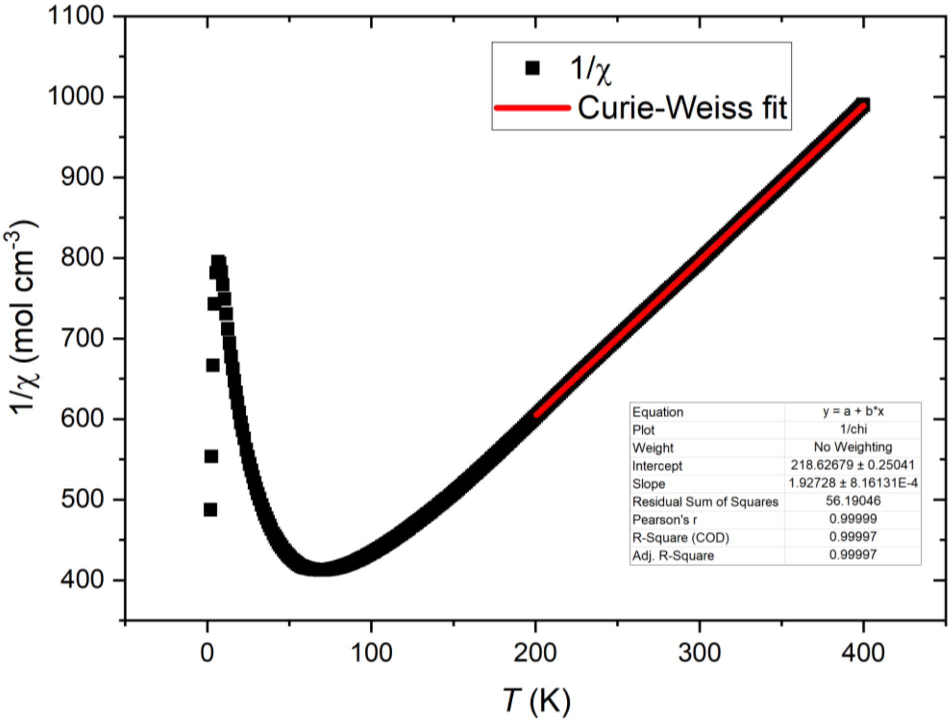

Figure S23. Curie-Weiss fit of $Ba_2CuTe_{0.9}W_{0.1}O_6$ (x = 0.9)

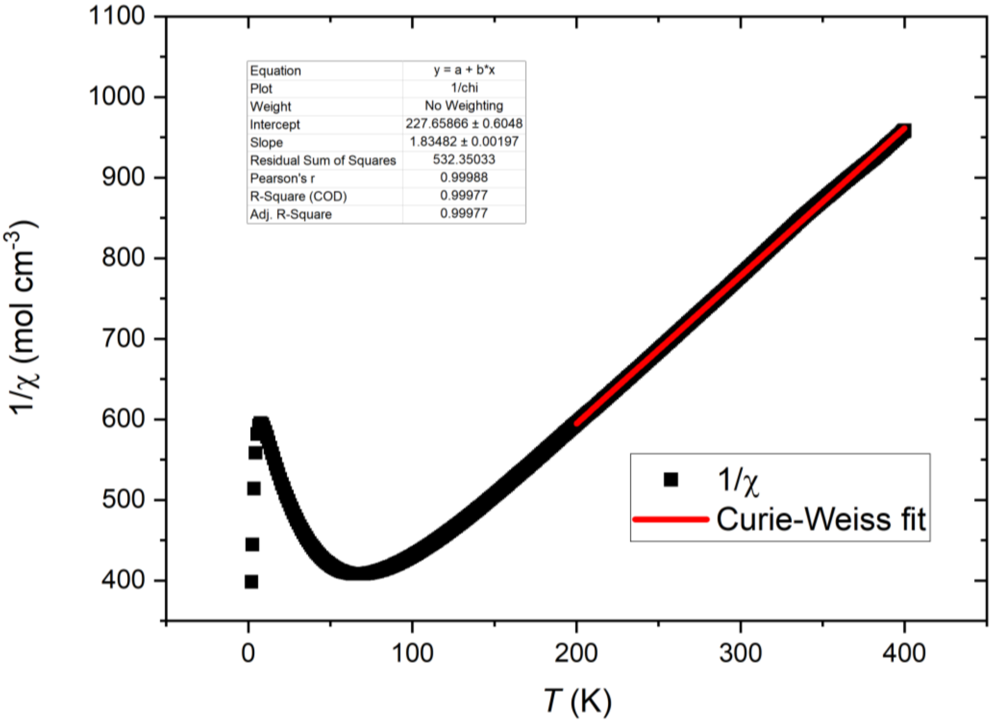

Figure S24. Curie-Weiss fit of $Ba_2CuTe_{0.8}W_{0.2}O_6$ (x = 0.8)

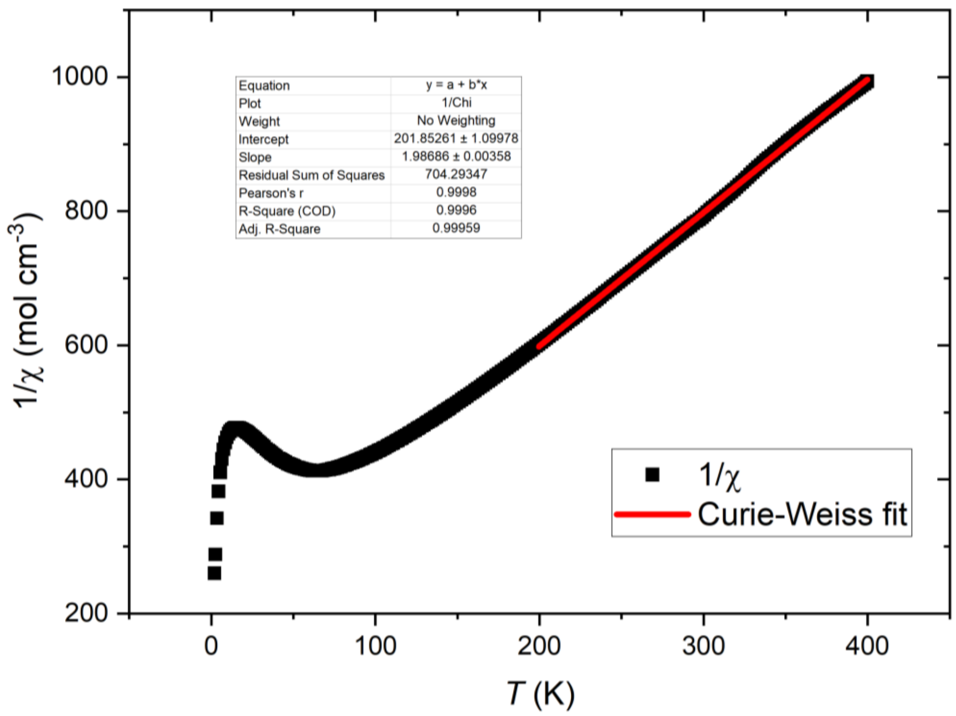

Figure S25. Curie-Weiss fit of $Ba_2CuTe_{0.7}W_{0.3}O_6$ (x = 0.7)

(b) Derivative of susceptibility curve

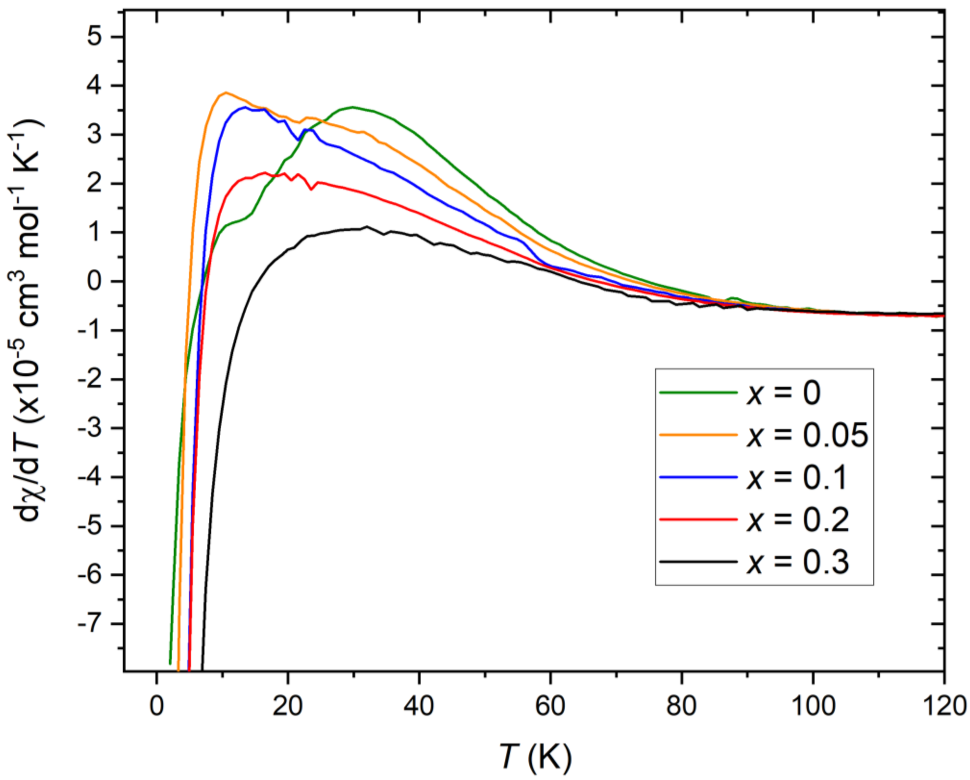

Figure S26. First derivative ($d\chi/dT$) of the χ vs T susceptibility data for all samples. No features suggesting possible magnetic ordering transitions are observed in any of the samples.

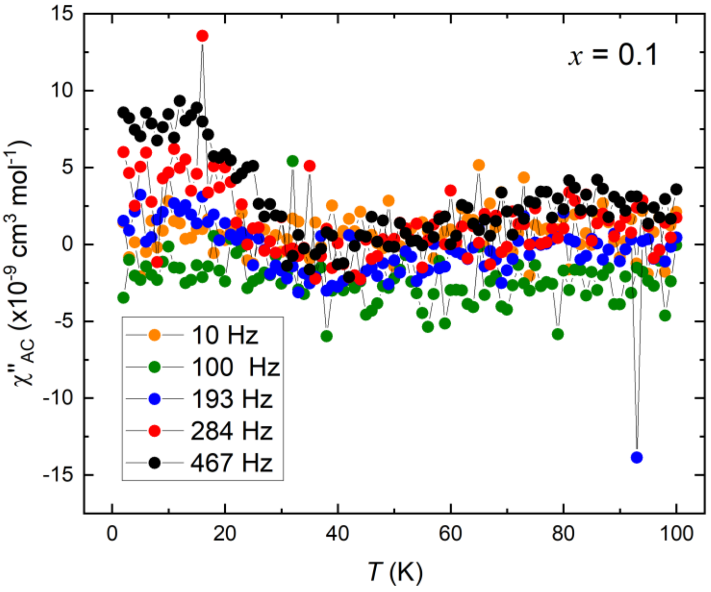

Figure S27. The imaginary component of the AC susceptibility χ_{AC}'' vs temperature, *T*, for $Ba_2CuTe_{0.9}W_{0.1}O_6$.

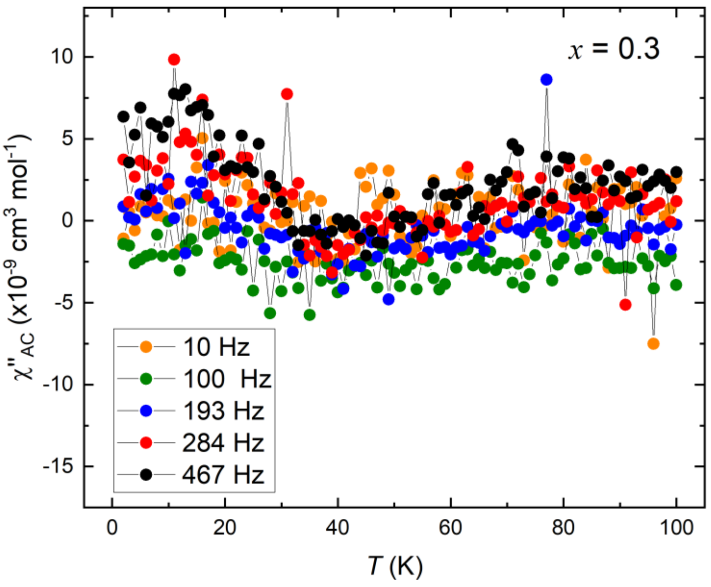

Figure S28. The imaginary component of the AC susceptibility χ_{AC}'' vs temperature, *T*, for $Ba_2CuTe_{0.7}W_{0.3}O_6$.

(c) Modelling the DC χ vs T susceptibility data using the spin chain and isolated two-leg spin ladder model

The expression for the Heisenberg spin chain model is shown below. Spin chain model fits to the 35-400 K χ vs T data were performed in Origin using two parameters, g and J_{chain} (in units of K for simplicity).[4,5] The fitting results are presented in Table S13. Graphical plots of the fits are provided in Figure S27.

$$\chi = \frac{N\mu_B^2}{k_B} \frac{0.25+0.14995\left(\frac{J_{chain}}{T}\right)+0.30094\left(\frac{J_{chain}}{T}\right)^2}{1+1.9862\left(\frac{J_{chain}}{T}\right)++0.68854\left(\frac{J_{chain}}{T}\right)^2+6.0626\left(\frac{J_{chain}}{T}\right)^3} + \chi_0 \quad (3)$$

Table S13: Result of spin chain model fitting to the Ba2CuTe1-xWxO6 35-400 K χ vs T data

Spin chain model	g	J_{chain} (K)	Goodness of fit
x = 0	2.27 ± 0.01	59.88 ± 0.35	0.99365
x = 0.05	2.181 ± 0.008	57.36 ± 0.25	0.99687
x = 0.1	2.145 ± 0.004	54.86 ± 0.12	0.99926
x = 0.2	2.084 ± 0.001	52.79 ± 0.041	0.9999
x = 0.3	2.044 ± 0.004	51.39 ± 0.14	0.99914

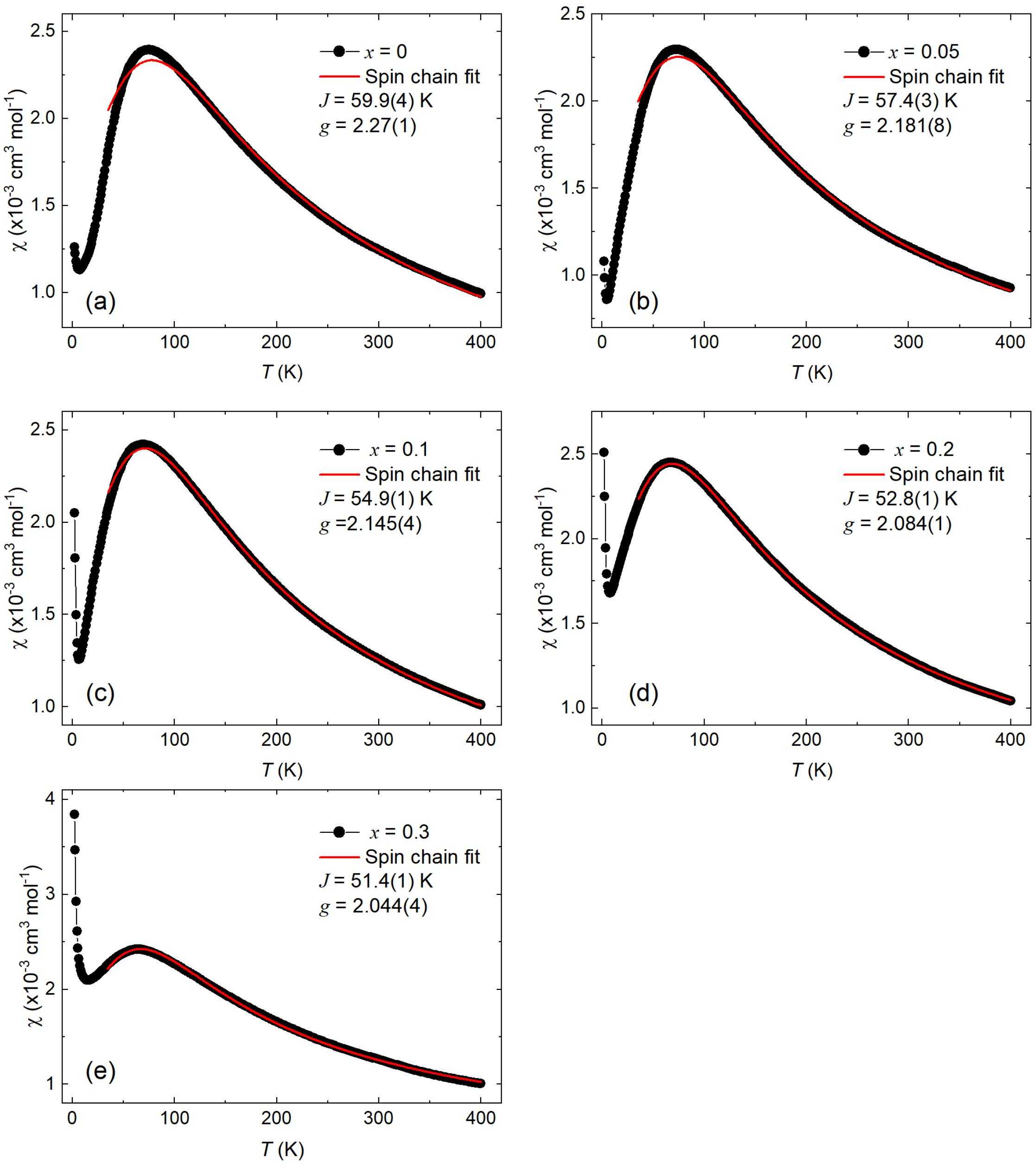

Figure S29. Graphical plots (a) to (e) showing the Heisenberg spin chain fits to the $Ba_2CuTe_{1-x}W_xO_6$ x = 0 to x = 0.3 DC susceptibility data, χ vs T, between 35 ≤ T ≤ 400 K. Within the legend of each plot, the g and J parameters are given, where J is expressed in units of Kelvin. The spin chain model (red line) provides a poor fit for the x = 0 sample in panel (a), but as x increases the fit improves. Upon reaching x = 0.3, the spin chain model provides a good description of the χ vs T data.

The susceptibility data were also fitted using the isolated two-leg spin ladder model. This model is an approximation based on fits to highly accurate QMC data of the isolated spin ladder model[6] (equations 17, A1 and 7). The molar magnetic susceptibility $\chi(T)$ can be described using a [6,6] Padé approximant. The model has the fitting parameters J_{leg}, J_{rung}/J_{leg} and g and it is valid for J_{rung}/J_{leg} ≤ 1. The molar magnetic susceptibility is given by:

$$\chi(T) = \frac{N_A g^2 \mu_B^2 e^{-\frac{\Delta J}{T}}}{4k_B T} P_6^6\left(\frac{T}{J_{leg}}\right) + \chi_0 \qquad (4)$$

where,

$$\Delta = 0.4030\left(\frac{J_{rung}}{J_{leg}}\right) + 0.0989\left(\frac{J_{rung}}{J_{leg}}\right)^3 \quad (5)$$

and

$$P_6^6\left(\frac{T}{J}\right) = \frac{1+\frac{N_1}{\left(\frac{T}{J_{leg}}\right)}+\frac{N_2}{\left(\frac{T}{J_{leg}}\right)^2}+\frac{N_3}{\left(\frac{T}{J_{leg}}\right)^3}+\frac{N_4}{\left(\frac{T}{J_{leg}}\right)^4}+\frac{N_5}{\left(\frac{T}{J_{leg}}\right)^5}+\frac{N_6}{\left(\frac{T}{J_{leg}}\right)^6}}{1+\frac{D_1}{\left(\frac{T}{J_{leg}}\right)}+\frac{D_2}{\left(\frac{T}{J_{leg}}\right)^2}+\frac{D_3}{\left(\frac{T}{J_{leg}}\right)^3}+\frac{D_4}{\left(\frac{T}{J_{leg}}\right)^4}+\frac{D_5}{\left(\frac{T}{J_{leg}}\right)^5}+\frac{D_6}{\left(\frac{T}{J_{leg}}\right)^6}} \quad (6)$$

and

$$N_n = N_{n0} + N_{1n1}\left(\frac{J_{rung}}{J_{leg}}\right) + N_{1n2}\left(\frac{J_{rung}}{J_{leg}}\right)^2 + N_{1n3}\left(\frac{J_{rung}}{J_{leg}}\right)^3 \quad (7)$$

and

$$D_n = D_{n0} + D_{1n1}\left(\frac{J_{rung}}{J_{leg}}\right) + D_{1n2}\left(\frac{J_{rung}}{J_{leg}}\right)^2 + D_{1n3}\left(\frac{J_{rung}}{J_{leg}}\right)^3 + D_{1n4}\left(\frac{J_{rung}}{J_{leg}}\right)^4 + D_{1n5}\left(\frac{J_{rung}}{J_{leg}}\right)^5 + D_{1n6}\left(\frac{J_{rung}}{J_{leg}}\right)^6 + D_{1n7}\left(\frac{J_{rung}}{J_{leg}}\right)^7 + D_{1n8}\left(\frac{J_{rung}}{J_{leg}}\right)^8 + D_{1n9}\left(\frac{J_{rung}}{J_{leg}}\right)^9 \quad (8)$$

with coefficients $N_n$and $D_n$from Table VII in ref [4]. Note that powers of J'/J up to 6 and 9 are only needed for D_2 with and D_3, respectively. J is expressed in units of K for simplicity.

The fitting results using the isolated two leg spin ladder model between 35 to 400 K are presented in Table S14. Graphical plots showing the spin ladder fits are provided in the main paper. Comparing the fits using the spin chain model in Figure S29 to the fits using the spin ladder model in Figure 6 of the paper shows the spin chain fit gradually improves as x increases.

Table S14: Result of isolated two-leg spin ladder model fitting to the Ba2CuTe1-xWxO6 35-400 K χ vs T data

Isolated two-leg spin ladder model	g	J_{leg} (K)	J_{rung}/J_{leg}	Goodness of fit
x = 0	2.2234 ± 0.0009	85.35 ± 0.042	1.0483 ± 5.9x10^{-4}	0.99994
x = 0.05	2.186 ± 0.002	91.95 ± 0.40	0.816 ± 0.0088	0.99985
x = 0.1	2.190 ± 0.001	98.84 ± 0.22	0.546 ± 0.0058	0.99994
x = 0.2	2.1360 ± 0.0005	102.76 ± 0.10	0.278 ± 0.0044	0.99999
x = 0.3	2.08 ± 0.02	102.37 ± 1.19	0.105 ± 0.14	0.99934

References

(1) Toby, B. H.; Von Dreele, R. B. GSAS-II: The Genesis of a Modern Open-Source All Purpose Crystallography Software Package. *J. Appl. Cryst.* **2013**, *46*, 544–549.

(2) Brown, I. D.; Altermatt, D. Bond-Valence Parameters Obtained from a Systematic Analysis of the Inorganic Crystal Structure Database. *Acta Cryst.* **1985**, *B41* (2), 244–247.

(3) Ravel, B.; Newville, M. ATHENA, ARTEMIS, HEPHAESTUS: Data Analysis for X-Ray Absorption Spectroscopy Using IFEFFIT. *J. Synchrotron Radiat.* **2005**, *12*, 537–541.

(4) Bonner, J. C.; Fisher, M. E. Linear Magnetic Chains with Anisotroyic Coupling. *Phys. Rev.* **1964**, *135*, A640-658.

(5) Hatfield, W. E.; Weller, R. R.; Hall, J. W. Exchange Coupling in the Sulfur-Bridged Quasi-Linear-Chain Compound Bis(Dimethyldithiocarbamato)Copper(II). Observations on Exchange in Sulfur-Bridged Copper(II) Compounds. *Inorg. Chemsitry* **1980**, *19*, 3825–3828.

(6) Johnston, D. C.; Troyer, M.; Miyahara, S.; Lidsky, D.; Ueda, K.; Azuma, M.; Hiroi, Z.; Takano, M.; Isobe, M.; Ueda, Y.; Korotin, M. A.; Anisimov, V. I.; Mahajan, A. V.; Miller, L. L. Magnetic Susceptibilities of Spin-1/2 Antiferromagnetic Heisenberg Ladders and Applications to Ladder Oxide Compounds. *arXiv:cond-mat/0001147 [cond-mat]* **2000**.
